# Research Progress of Natural Active Substances with Immunosuppressive Activity

**DOI:** 10.3390/molecules29102359

**Published:** 2024-05-16

**Authors:** Fei Shao, Qiying Shen, Zhengfei Yang, Wenqian Yang, Zixiang Lu, Jie Zheng, Liming Zhang, Hangying Li

**Affiliations:** 1 School of Pharmacy, Ningxia Medical University, Yinchuan 750004, China; shaofei433@163.com (F.S.);; 2 School of Traditional Chinese Medicine, Ningxia Medical University, Yinchuan 750004, China; 3 Key Laboratory of Craniocerebral Diseases, Ningxia Medical University, Yinchuan 750004, China

**Keywords:** immunosuppressive activity, chemical structures, source, structure-activity relationship

## Abstract

The increasing prevalence of autoimmune diseases globally has prompted extensive research and the development of immunosuppressants. Currently, immunosuppressive drugs such as cyclosporine, rapamycin, and tacrolimus have been utilized in clinical practice. However, long-term use of these drugs may lead to a series of adverse effects. Therefore, there is an urgent need to explore novel drug candidates for treating autoimmune diseases. This review aims to find potential candidate molecules for natural immunosuppressive compounds derived from plants, animals, and fungi over the past decade. These compounds include terpenoids, alkaloids, phenolic compounds, flavonoids, and others. Among them, compounds **49**, **151**, **173**, **200**, **204**, and **247** have excellent activity; their IC_50_ were less than 1 μM. A total of 109 compounds have good immunosuppressive activity, with IC_50_ ranging from 1 to 10 μM. These active compounds have high medicinal potential. The names, sources, structures, immunosuppressive activity, and the structure-activity relationship were summarized and analyzed.

## 1. Introduction

Autoimmune diseases are characterized by the immune system attacking its antigens [1], which include psoriasis, rheumatoid arthritis (RA), systemic lupus erythematosus (SLE), and related disorders. Currently, approximately 11–15% of the population is affected by autoimmune diseases [2]. An estimated 60 million people have psoriasis worldwide [3], and the global prevalence of RA ranges from 0.23% to 0.36% [4], while the prevalence of SLE is 0.24% with a 10-year survival rate of 56.5–98.2% [5]. More than 80 autoimmune diseases were identified in humans, affecting various organs and systems such as the skin, kidneys, blood vessels, brain, and joints [6,7]. Therefore, effective treatment of autoimmune diseases is crucial.

The pathogenesis of autoimmune diseases may be related to factors of genetics, environment, immune tolerance damage, and abnormal regulation of immune responses [8,9]. For the treatment of autoimmune diseases, the commonly used clinical treatment method is immunosuppressive drugs [10,11]. Immunosuppressants can suppress aberrant immune phenomena by inhibiting the proliferation and function of immune response-related cells (mainly T-cells and B-cells) [12,13,14,15,16]. It is mainly employed for the prevention and treatment of graft rejection and autoimmune diseases [17]. There is the fact that immunosuppressants play a crucial role in suppressing abnormal immune responses, and the new immunosuppressants need to be explored [18,19,20].

Many immunosuppressants from natural sources have been used in clinical practice, such as mycophenolate mofetil (MMF), rapamycin (RPM), and tacrolimus. The MMF was derived from *Penicillium* mold fermentation [21,22]. RPM is a lactone derived from the fermentation broth of *Streptomyces hygroscopicus* [23]. Tacrolimus, a 23-membered macrolide antibiotic, was produced through fermentation by *Streptomyces tsukubaensis* [24]. This review examines natural products with immunomodulatory properties, including terpenoids, alkaloids, phenolic compounds, flavonoids, and others, from 2013 to 2024. The names, structures, sources, and activity of natural products with immunosuppressive activity are systematically and comprehensively summarized. The structure-activity relationship of compounds with significant immunosuppressive activity is discussed. Additionally, models for screening immunosuppressive activity are also summarized.

## 2. The Immunosupperssive Activity of Animal and Cell Models

Seven cellular and six animal models are commonly used for screening immunosuppressive activity, as shown in Table 1 and Table 2. Selected animals included Balb/c mice, ICR mice, C57BL/6 mice, Wistar rats, and Swiss albino mice. The drugs utilized for animal modeling include cyclophosphamide, carbon tetrachloride (CCl_4_), pristane, delayed-type hypersensitivity (DTH), sheep red blood cells (SRBC), and xylene. For the cell models, concanavalin A (ConA) and lipopolysaccharide (LPS) are primarily employed in conjunction with human mononuclear cells, splenocytes, dendritic cells, BV-2 microglia, and RAW264.7 macrophages. In this review, the immunosuppressive activity of each compound is examined mainly using different cell models.

## 3. Natural Products with Immunosuppressive Activity

Natural products, due to their diverse activities and unique structures, offer an inexhaustible source for the discovery of small-molecule drug leads. It is expected to discover non-toxic and highly bioavailable immunosuppressants from natural products [25]. Numerous studies have demonstrated the immunosuppressive activity of natural product extracts containing terpenoids, alkaloids, phenolic compounds, flavonoids, and others [26].

### 3.1. Terpenoids

Terpenoids are a class of naturally occurring products with diverse structures that have been used by humans in various fields such as food, pharmaceuticals, and chemical industries [27]. Some sesquiterpenoids, diterpenoids, and triterpenoids exhibit immunosuppressive activity.

#### 3.1.1. Sesquiterpenoids

**Figure 1:**
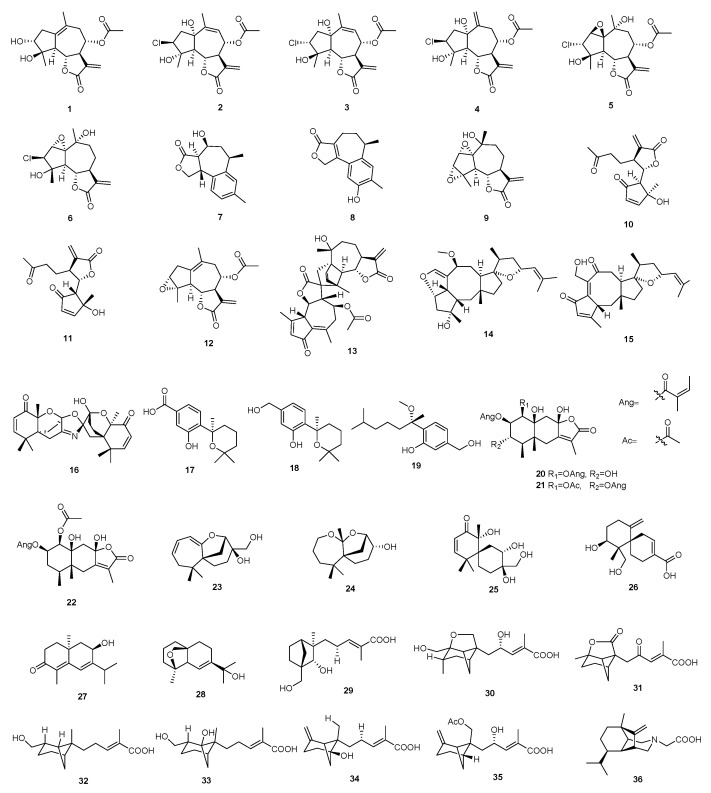
Structures of sesquiterpenoids **1**–**36**.

There are 36 sesquiterpenoids with immunosuppressive activity, and their structures are shown in Figure 1. The sources, selected models, and immunosuppressive activities are shown in Table S1.

Six sesquiterpenoids (**1**–**6**) with immunosuppressive activity were isolated from *Artemisia argyi*, named argyinolide G (**1**), 8*α*-acetoxy-3*β*-chloro-1*α*,4*α*-dihydroxyguai-9,11(13)-dien-6*α*,12-olide (**2**), 8α-acetoxy-3*α*-chloro-1*α*,4*β*-dihydroxyguai-9,11(13)-dien-6*α*,12-olide (**3**), 8*α*-acetoxy-3*β*-chloro-1*α*,4*α*-dihydroxyguai-10(14),11(13)-dien-6*α*,12-olide (**4**), 8*α*-acetoxy-3*α*-chloro-1*β*,2*β*-epoxy-4*β*,10*α*-dihydroxy-5*α*,7*α*H-guai-11(13)-en-12,6*α*-olide (**5**), and 3*β*-chloro-1*α*,2*α*-epoxy-4*α*,10*α*-dihydroxy-5*α*,7*α*H-guai-11(13)-en-12,6*α*-olide (**6**). Compounds **1**–**6** exhibited potent inhibition of NO production in LPS-stimulated BV-2 microglia, with IC_50_ values of 5.3, 3.2, 6.9, 4.2, 22.2, and 6.4 μM, respectively [28].

Tremutins A and B (**7** and **8**) were isolated from the basidiomycetes *Irpex lacteus*. Compound **7** inhibited the B lymphocyte cells with an IC_50_ value of 22.4 μM. Meanwhile, compound **8** inhibited T-cell proliferation and B-cell proliferation with IC_50_ values of 16.7 and 13.6 μM, respectively [29]. Canin (**9**), *seco*-tanapartholide B (**10**), *seco*-tanapartholide A (**11**), arteglasin A (**12**), and 8-acetylarteminolide (**13**) were isolated from the ethyl acetate extract of *A. argyi*. Compounds **9**–**13** showed significant inhibition of T lymphocyte proliferation, with IC_50_ values of 2.7, 1.0, 1.2, 1.9, and 3.2 μM, respectively [28]. Maydispenoids A and B (**14** and **15**) were isolated from *bipolaris maydis*, which exhibited inhibitory effects on murine splenocyte proliferation stimulated by anti-CD3/anti-CD28 monoclonal antibodies (mAbs), with IC_50_ values of 5.28 and 9.38 μM [30]. A sesquiterpene named antroxazole A (**16**) was isolated from *Epigynum griffithianum.* Compound **16** was evaluated for its immunosuppressive activity on the proliferation of LPS-induced B-cells, with an IC_50_ value of 16.3 μM [14]. Three sesquiterpenoids, named (+)-aspersydowin A (7*S*) (**17**), (+)-aspersydowin B (7*S*,11*S*) (**18**), and (7*S*)-(+)-7-*O*-methylsydonol (**19**), were isolated from *Aspergillus sydowii*. Compounds **17**–**19** inhibited the proliferation of ConA-induced T-cells with an IC_50_ value greater than 40 μM. Compounds **17**–**19** inhibited the proliferation of LPS-induced B-cells, with IC_50_ values of 10.9, 17.6, and 13.4 μM, respectively [31].

Parasubolide D (**20**), parasubolide E (**21**), and parasubolide L (**22**) were isolated from the plant *Parasenecio albus*. Compounds **20**–**22** had immunosuppressant activity against B-cell proliferation with IC_50_ values of 23.1, 33.8, and 26.6 μM, respectively [32]. Four sesquiterpenoids, named steccherins A–D (**23**–**26**), were extracted from *Steccherinum ochraceum.* Compounds **23**–**26** inhibited the T and B lymphocytes with IC_50_ values ranging from 37.8 to over 40 μM and 6.2 to over 40 μM, respectively [33].

Soltorvum A and soltorvum B (**27** and **28**), obtained from the stems of *Solanum torvum*, could inhibit the activity of ConA-induced splenocyte proliferation and possessed IC_50_ values of 27 and 18 μM, respectively [34]. Craterodoratin C (**29**), craterodoratin J (**30**), craterodoratins L–O (**31**–**34**), craterodoratin Q (**35**), and craterodoratin S (**36**) were isolated from the edible mushroom *Craterellus ordoratus*. Compounds **29**–**34** and **36** exhibit potent inhibitory activity against B lymphocyte cells, with IC_50_ values ranging from 12.62 to 22.68 μM. Compound **35** inhibited the ConA-induced proliferation of T lymphocyte cells with an IC_50_ value of 31.50 μM [35].

#### 3.1.2. Diterpenoids

**Figure 2:**
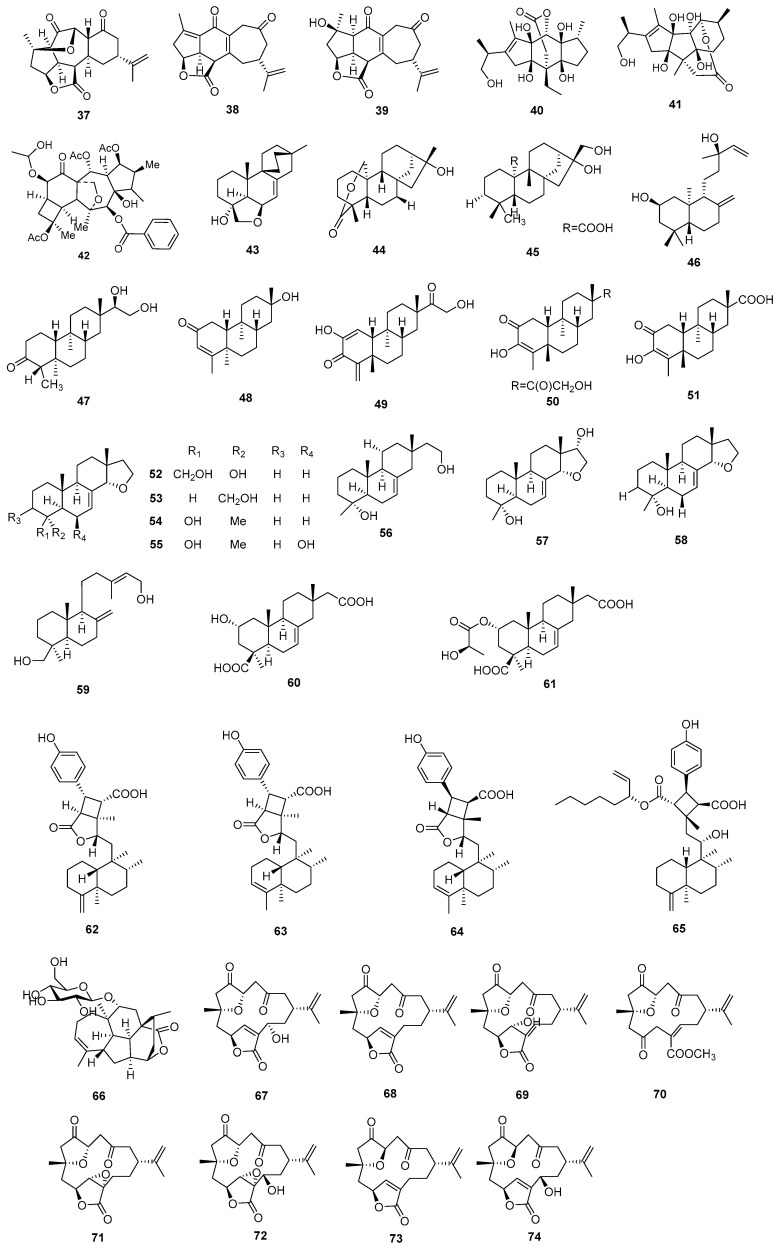
Structures of diterpenoids **37**–**74**.

**Figure 3:**
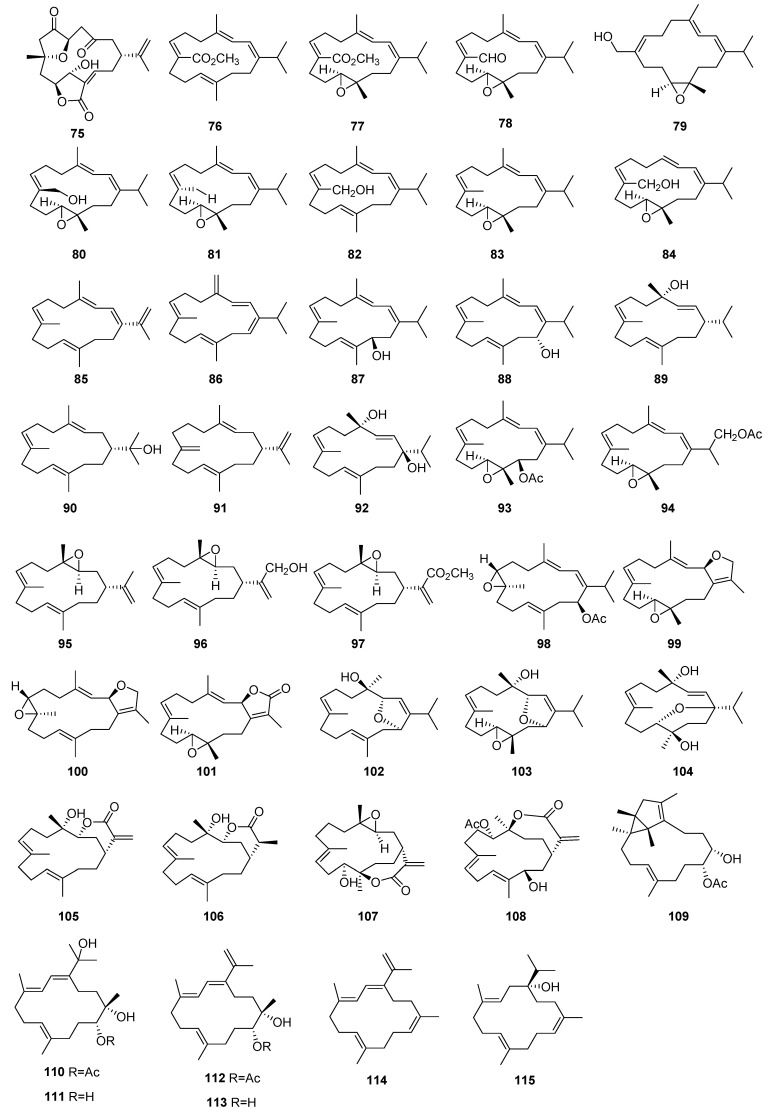
Structures of diterpenoids **75**–**115**.

The structures, sources, selected models, and immunosuppressive activities of 79 diterpenoids are summarized in Figure 2 and Figure 3 and Table S2. 

Three diterpenoids, named ineleganolide (**37**), yonarolide (**38**), and scabrolide A (**39**), were isolated from the corals *Sinularia scabra* and *Sinularia polydactyla*. Compounds **37**–**39** demonstrated significant inhibitory effects on the proliferation of T and B-cells, with an IC_50_ value exceeding 50 μM [36]. Cinncassiol G and cinnacasol (**40** and **41**) were isolated from the stem bark of *Cinnamomum cassia*. Compounds **40** and **41** inhibited the proliferation of ConA-induced T-cells and LPS-induced B-cells. The proliferation of T-cells was significantly suppressed by both compounds at a concentration of 50 μM. Compound **40** exhibited an inhibition rate of 86.1%, while compound **41** showed a slightly lower inhibition rate of 58.8% [37].

A new diterpenoid with a five-membered ring, named 3,5,10-*O*-triacetyl-8- -isobutanoyl-14-*O*-benzoylcyclomyrsinol (**42**), was isolated from *Euphorbia kopetdaghi* Prokh. Compound **42** showed significant activity against phytohemagglutinin-activated T-cell proliferation, with an IC_50_ of 1.83 mg/mL [38]. Xylarilongipin A (**43**) was extracted from the culture broth of the fungicolous fungus *Xylaria longipes* HFG1018, which inhabits the medicinal fungus *Fomitopsis betulinus*. Compound **43** exhibited moderate inhibitory effects on the proliferation of T and B lymphocytes, with IC_50_ values measured at 13.6 μM and 22.4 μM, respectively [39].

Three diterpenoids, named tripterifordin (**44**), 16*α*,17-dihydroxy-*ent*-kaur-20-al (**45**), and *ent*-2*β*-hydroxymanool (**46**), were isolated from *Ligularia fischeri.* Compounds **44**–**46** had moderate inhibitory activity against human B lymphoblast HMy2. CIR cells with IC_50_ values of 56.3, 13.3, and 31.4 μM, respectively [40]. There were five diterpenoids (**47**–**51**) with immunosuppressive activity from *Koilodepas hainanense*, named koilodenoid D (**47**), koilodenoid G (**48**), *ent*-5*α*,2,15-dioxodolabr-3-ene-3,16-diol (**49**), *ent*-5*α*,3,15-dioxodolabr-1,4(18)-diene-2,16-diol (**50**), and *ent*-16-nor-5*α*,13*α*-2-oxodolabra-3-en-3-ol-15-oic acid (**51**). Compounds **47**–**51** inhibited T-cell and B-cell with IC_50_ values ranging from 0.86 to 30.54 μM and 0.29 to 10.2 μM, respectively [41].

Eight diterpenoids were extracted from cultures of the fungicolous fungus *Xylaria longipes* HFG1018: xylarinorditerpene B (**52**), xylarinorditerpene C (**53**), xylarinorditerpene D (**54**), xylarinorditerpene E (**55**), xylarinorditerpene I (**56**), xylarinorditerpene N (**57**), 14*α*,16-epoxy-18-norisopimar-7-en-4*α*-ol (**58**), and agatadiol (**59**). Compounds **52**–**59** inhibited T-cell and B-cell proliferation with IC_50_ values ranging from 1.0 to 27.2 μM and 16.1 to 51.8 μM, respectively. DXMS (dexamethasone) was selected as the positive control against T-cell and B-cell proliferation with IC_50_ values of 1.6 and 0.8 μM, respectively [42].

Robustaditerpenes C and E (**60** and **61**) were isolated from cultures of the endophytic fungus *Ilyonectria robusta*. Compound **60** effectively inhibited the proliferation of B lymphocytes, with an IC_50_ value of 17.42 μM. Similarly, compound **61** showed significant inhibition of the proliferation of T lymphocytes, with an IC_50_ value of 75.22 μM [43]. Three diterpenoids were isolated from *Isodon scoparius,* named scopariusicide I (**62**), scopariusicide J (**63**), and scopariusicide L (**64**). Compounds **62**–**64** demonstrated significant suppression of murine splenocyte proliferation when stimulated with anti-CD_3_/anti-CD_28_ monoclonal antibodies, with IC_50_ values ranging from 9.4 to 16.3 μM [44]. Scopariusic acid (**66**), a ent-clerodane-based meroditerpenoid with a unique cyclobutane ring, was isolated from the aerial parts of *Isodon scoparius*. Compound **65** showed significant activity against T-cell proliferation with an IC_50_ value of 2.6 μM [45]. A new diterpenoid named ceforloid F (**65**) was isolated from *Cephalotaxus fortunei* var. *alpina* and *C. sinensis*. Compound **66** exhibited noteworthy efficacy in inhibiting the proliferation of T-cells, with an IC_50_ value of 1.93 μM [46].

Nine diterpenoids, named xiguscabrolide H (**67**), 10-*epi*-gyrosanolide E (**68**), 5-*epi*-sinuleptolide (**69**), norcembrene (**70**), scabrolide D (**71**), scabrolide G (**72**), sinularcasbane O (**73**), gyrosanolide F (**74**), and sinuleptolide (**75**), were extracted from the corals *Sinularia scabra* and *Sinularia polydactyla.* Compounds **67**–**75** inhibited the T and B lymphocytes with IC_50_ values ranging from 8.5 to over 50.00 μM and 20.5 to over 50.00 μM, respectively [36].

Compounds **76**–**108** were extracted from the coral *S. scabra*, whose names are shown in Table S2. The bioassay revealed that several isolates displayed inhibitory effects on the proliferation of T lymphocytes and B lymphocytes. Compounds **76**–**108** inhibited T-cell and B-cell proliferation with IC_50_ values ranging from 4.5 to over 50.0 μM and 4.4 to over 50.0 μM, respectively [47].

Compounds **109**–**115** were extracted from the Hainan Soft Coral *Sarcophyton mililatensis*, whose names were shown in Table S2. The immunosuppressive activity of compounds **109**–**115** was tested, which indicated these compounds had moderate activities against T-cell and B-cell proliferation with IC_50_ values ranging from 11.4 to greater than 50 μM and 4.8 to greater than 50.0 μM, respectively [48].

#### 3.1.3. Triterpenoids

**Figure 4:**
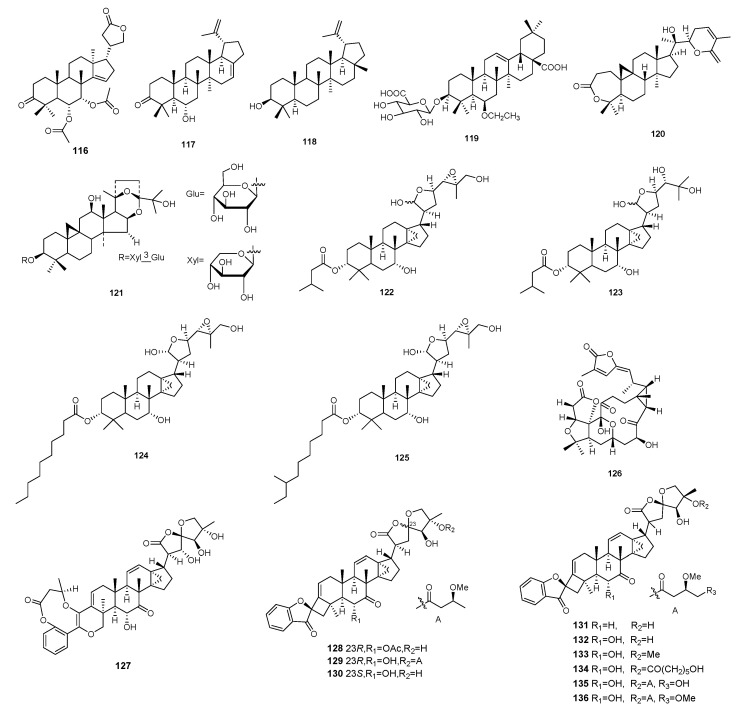
Structures of triterpenoids **116**–**136**.

There are 21 triterpenoids with promising immunosuppressive activity. Table S3 and Figure 4 summarize the sources, structures, selected models, and immunosuppressive activities of these compounds.

A triterpene, named munronoid P (**116**), was discovered in the aerial parts of *Munronia pinnata*. Compound **116** demonstrated moderate inhibitory effects on T-cell proliferation, with an IC_50_ value of 2.73 μM. Additionally, it also exhibited inhibition against B-cell proliferation, with an IC_50_ value of 34.88 μM [49]. Triterhyper A and lupeol (**117** and **118**) were extracted from *Hypericum longistylum*. These compounds showed inhibitory effects on the growth of murine splenocytes stimulated by anti-CD3/anti-CD28 monoclonal antibodies (mAbs) and LPS, with IC_50_ values of 4.56 μM and 18.34 μM, respectively [50]. A new triterpenoid saponin (**119**) was isolated from *Epigynum griffithianum.* Compound **119** was evaluated in vitro for its immunosuppressive activity on the proliferation of mouse splenocytes, with an IC_50_ value of 25 μM. ^14^Schincarin C (**120**) was isolated from the plants of the genus *Schisandra*, which showed immunosuppressive activities against the proliferation of the T and B-cells with IC_50_ values of 10.21 and 5.83 μM, respectively [51]. BC-1 (**121**) was isolated from *Beesia calthaefolia*, which inhibited murine T lymphocyte proliferation with IC_50_ values of 9.8 μM [52]. Dictabretols A–D (**122**–**125**) were isolated from the root bark of *Dictamnus dasycarpus* using guided fractionation. An antiproliferative assay on T-cells using splenocytes was performed in vitro. Compounds **122**–**125** were evaluated for their immunosuppressive activity on T-cells and demonstrated inhibition of proliferation of activated T-cells, with IC_50_ values of 1.5, greater than 20, 1.8, and 1.5 μM, respectively [53]. Compound **126**, named schincalactones A, was isolated from *Schisandra.* Compound **126** had certain immunosuppressive activity against LPS-induced B-cells and ConA-induced T-cells, with IC_50_ values of 36.84 and over 50.91 μM, respectively [54].

Four triterpenoids were isolated from *Phyllanthus hainanensis*, including phainanolide A (**127**) and phainanoid G (**128**); phainanoid H (**129**); and phainanoid I (**130**). The immunosuppressive activity of compounds **127**–**130** was tested, which indicated these compounds had moderate activities against T-cell and B-cell proliferation with IC_50_ values ranging from 16.15 to 566.83 μM and 8.24 to 456.63 μM, respectively [55]. Phainanoids A–F (**131**–**136**), six highly modified triterpe-noids with a new carbon skeleton, were isolated from *Phylanthus hainanensis*. The immunosuppressive activity of compounds **131**–**136** was tested, which indicated these compounds had moderate activities against T-cell and B-cell proliferation with IC_50_ values ranging from 2.04 to 192.8 μM and less than 1.60 to 249.49 μM, respectively [56].

#### 3.1.4. Other Terpenoids

**Figure 5:**
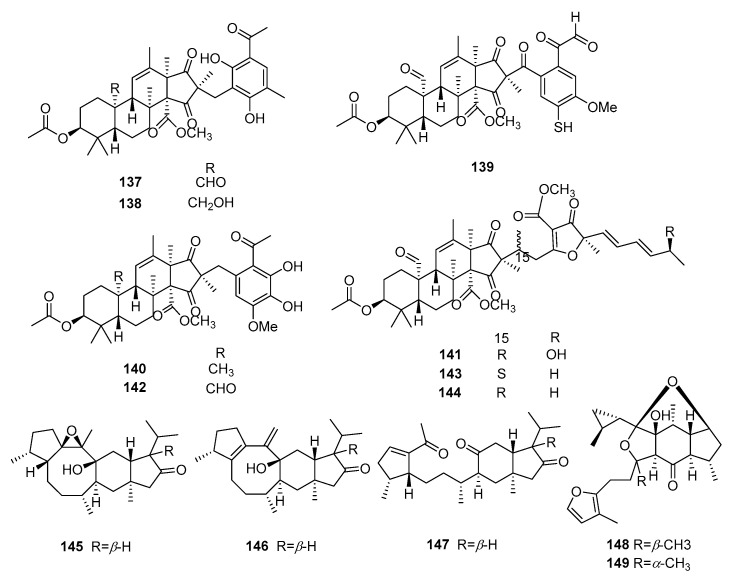
Structures of other terpenoids **137**–**149**.

There are thirteen other triterpenoids with promising immunosuppressive activity. Table S4 and Figure 5 summarize the sources, structures, selected models, and immunosuppressive activities of these compounds.

Peniandranoids A (**137**), peniandranoids B (**138**), peniandranoids C (**139**), peniandranoids D (**140**), peniandranoids E (**141**), isopenicin A (**142**), isopenicin B (**143**), and isopenicin C (**142**–**144**) were isolated from the fermentation of a soil-derived fungus, *Penicillium* sp. sb62. Compounds **137**–**144** exhibited potent immunosuppressive effects on T-cell proliferation, with EC_50_ values ranging from 4.3 to 27 μM. Similarly, they also demonstrated strong immunosuppressive activities against B-cell proliferation, with EC_50_ values ranging from 7.5 to 23 μM [56]. Three gentianellane-type sesterterpenoids named nitidasin (**145**), gentianelloid F (**146**), and alborosin (**147**) were isolated from whole plants of *Gentianella turkestanorum*, which showed moderate to significant immunosuppressive activity against anti-CD3/anti-CD28 monoclonal antibodies (mAbs), with IC_50_ values of 12.31, 13.68, and 14.31 μM [57].

A pair of C-14 epimeric sesterterpenoids, colquhounoid D (**148**) and 14-*epi*-colquhounoid D (**149**), were isolated from *Colquhounia coccinea* var. mollis. Compounds **148** and **149** showed significant immunosuppressive effects on the cytokine IFN-*γ* secretion of mouse splenocytes induced by anti-CD3/CD4, with IC_50_ values of 8.38 μM and 5.79 μM, respectively [58].

### 3.2. Alkaloids

**Figure 6:**
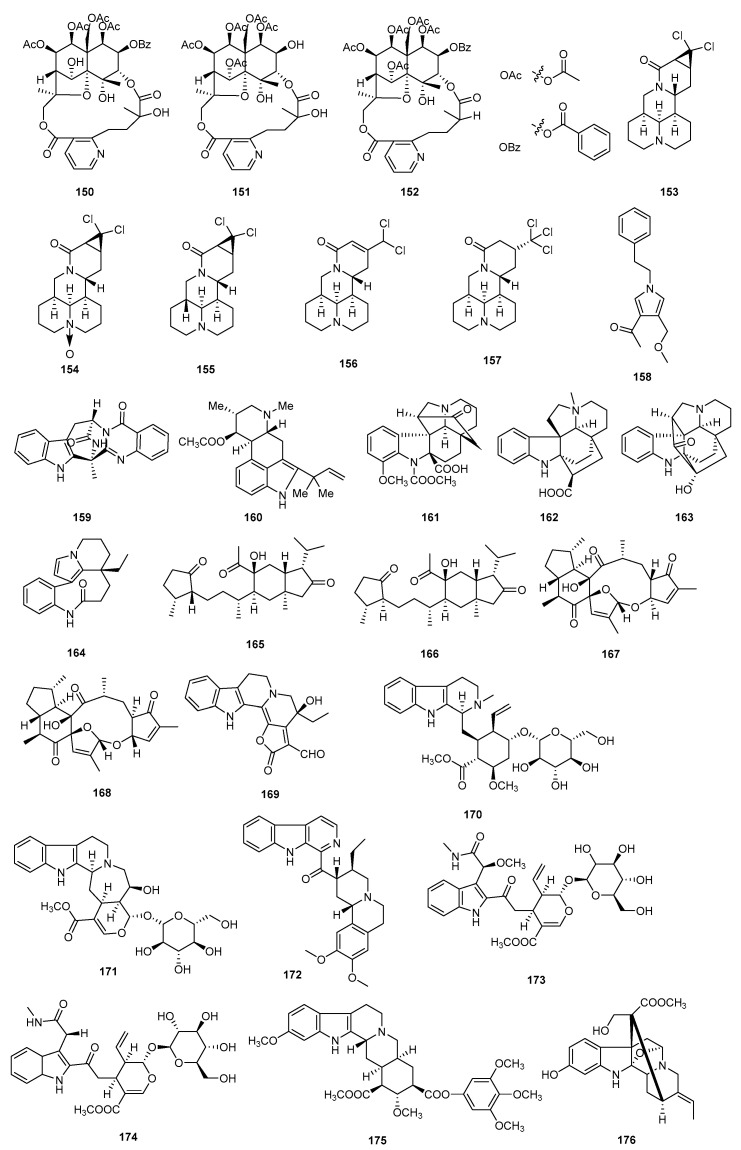
Structures of alkaloids **150**–**176**.

Alkaloids constitute a group of nitrogen-containing organic compounds [59]. Over the last ten years, several alkaloids have shown immunosuppressive activity and have been extracted from natural sources. The structures, sources, selected models, and immunosuppressive activities of 27 alkaloids are shown in Figure 6 and Table S5.

Three alkaloids named wilfordatin E (**150**), tripfordine A (**151**), and wilforine (**152**) were isolated from the roots of *Tripterygium wilfordii* Hook. The inhibitory effects of compounds **150**–**152** on the nuclear factor-kappa B pathway were assessed in HEK293 cells induced with LPS. The IC_50_ values obtained for these compounds were 8.75 μM, 0.74 μM, and 15.66 μM, respectively [60]. Alopecines A–E (**153**–**157**) were isolated from the seeds of *Sophora alopecuroides*, which showed moderate to significant immunosuppressive activity against T-cell and B-cell proliferation, with the IC_50_ values ranging from 3.9 to 100 μM and from 3.7 to 96.8 μM, respectively [61]. 

A pyrrole alkaloid, albifpyrrols B (**158**), was isolated from the endophytic fungus *Albifmbria viridis* collected. Compound **158** exhibits inhibition against B lymphocyte cells with an IC_50_ value of 16.16 μM [62]. Fumiquinazoline J and fumigaclavine C (**159** and **160**) were isolated from mangrove-derived *Aspergillus fumigatus* HQD24. Compound **159** effectively suppressed the proliferation of T lymphocytes induced by ConA and B lymphocytes induced by LPS, with IC_50_ values of 29.38 μM and 162.58 μM, respectively. Compound **160** remarkable inhibited proliferation of ConA-induced T with an IC_50_ value of 53.12 μM [63]. 

Four alkaloids (**161**–**164**) were isolated from *Kopsia officinalis*, named 12-methoxychanofruticosinic acid (**161**), *N-*(4)-methylkopsininate (**162**), demethoxycarbonylkopsin (**163**), and rhazinilam (**164**). Compounds **161**–**164** have immunosuppressive activity on human T-cell proliferation with IC_50_ values of 27.8, 21.6, 25.4, and 1.0 μM, respectively [64]. 

Two sesterterpenoids, gentianelloids A and B (**165** and **166**), which possess an unusual seco-gentianellane skeleton, were discovered in the traditional Uighur medicine of *Gentianella turkestanorum*. Compounds **165** and **166** exhibited significant immunosuppressive activity against the proliferation of murine splenocytes activated by anti-CD3/anti-CD28 monoclonal antibodies, with IC_50_ values of 5.64 and 3.93 μM, respectively [65]. Two novel diastereomeric sesterterpenoids, eurysoloids A (**167**) and B (**168**), were discovered in *Eurysolen gracilis* Prain. These compounds possess a unique pentacyclic structure with an extraordinary macrocyclic ether system. Compounds **167** and **168** exhibited immunosuppressive activity by effectively inhibiting the production of the cytokine IFN-*γ* in T-cells, with IC_50_ values of 17.4 and 15.94 μM, respectively [66].

A monoterpenoid indole alkaloids named rhazinilam (**169**) was obtained from the bark of *Pausinystalia yohimbe*. Compounds **169** exhibited moderate inhibition against ConA-induced T lymphocyte proliferation, with IC_50_ values of 16.8 μM. Additionally, they showed moderate inhibition against LPS-induced B lymphocyte proliferation, with IC_50_ values of 13.5 μM [67].

Three compounds, named ophiorrhine C (**170**), ophiorrhine D (**171**), and ophiorrhine E (**172**), were obtained from the plant *Ophiorrhiza cantoniensis* Hace. Compounds **170**–**172** showed immunosuppressive activities against the proliferation of T and B lymphocytes, with IC_50_ values from 17.9 to over 200 μM and 8.7 to 116.2 μM, respectively [68]. Two alkaloids, named ophiorrhine F (**173**) and G (**174**), were obtained from the aerial parts of *Ophiorrhiza japonica*. Both compounds demonstrated significant inhibition of B-cell proliferation induced by LPS, with IC_50_ values of 0.38 and 47.37 μM, respectively. CsA was employed as a positive control to assess its inhibitory effects on T-cell proliferation induced by ConA and B-cell proliferation induced by LPS, with IC_50_ values of 0.03 and 0.32 μM, respectively [69]. 11-Hydroxyburnamine (**175**) and reserpine (**176**) were extracted from the total alkaloid extract of Rauvolfia yunnanensis. These compounds have shown immunosuppressive effects on human T-cell proliferation, with IC_50_ values of 5.9 and 5.0 μM, respectively [70].

### 3.3. Phenolic Compounds

**Figure 7:**
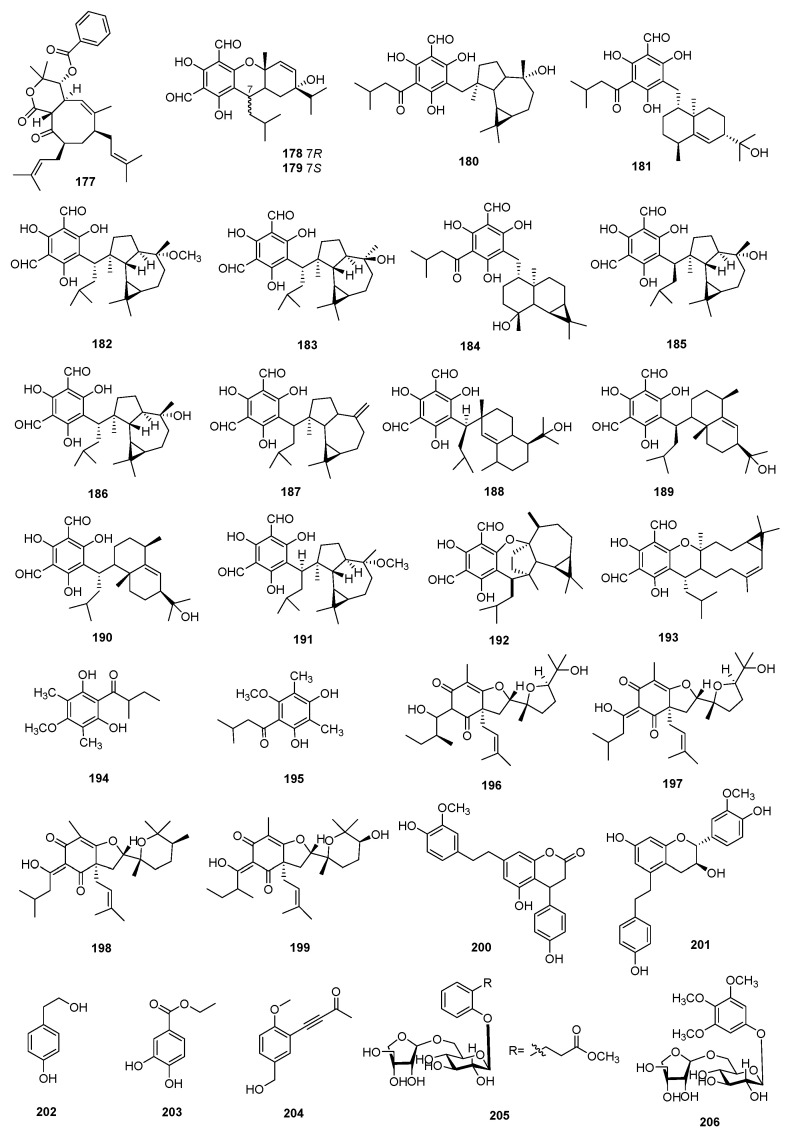
Structures of phenolic compounds **177**–**206**.

**Figure 8:**
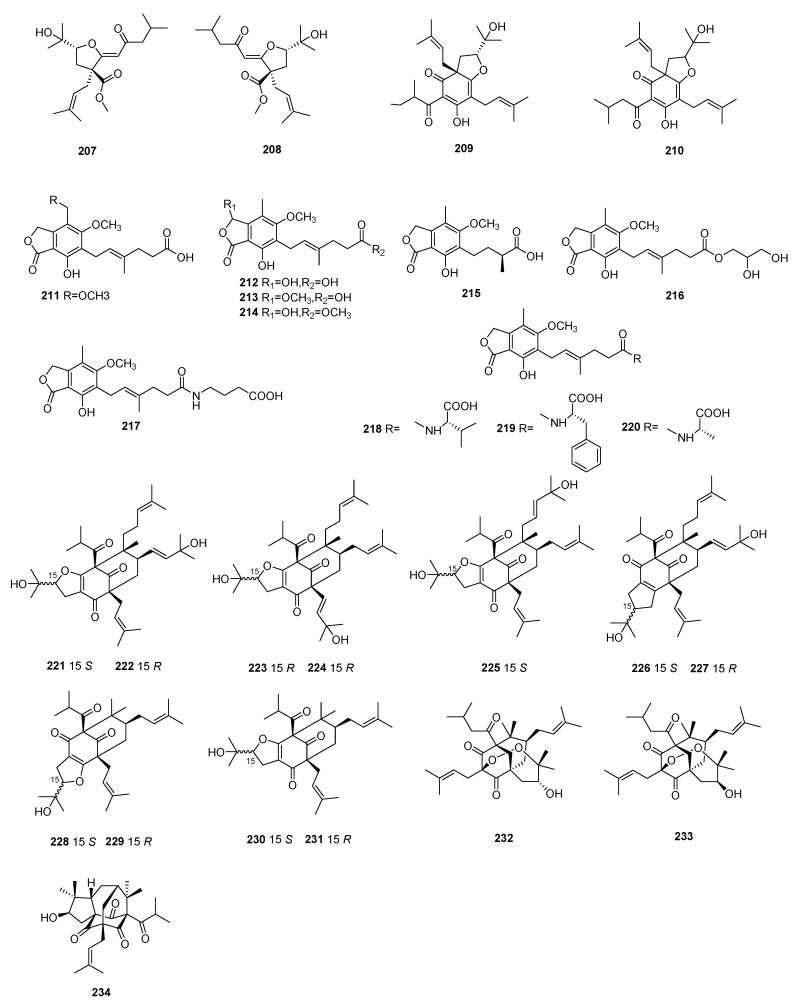
Structures of phenolic compounds **207**–**234**.

In recent years, phenolic compounds have been discovered to possess immunosuppressive activity, rendering them potential candidates for the prevention and treatment of autoimmune diseases [71]. The structures, sources, selected models, and immunosuppressive activities are shown in Figure 7 and Figure 8 and Table S6.

Some of the resorcinols also had a significant immunosuppressive effect. Compound **177**, named hypaluton A, was isolated from *Hypericum patulum* and had inhibitory activity against B lymphocyte proliferation with an IC_50_ value of 6.86 μM [72]. Eighteen phloroglucinol derivatives were isolated from the fruits of *Eucalyptus globulus*, named eucalyptin A (**178**), eucalyptin B (**179**), eucalyptin C (**180**), eucalyptin D (**181**), eucalyptin E (**182**), eucalyptin F (**183**), eucalyptin G (**184**), macrocarpal A (**185**), macrocarpal B (**186**), macrocarpal C (**187**), macrocarpal D (**188**), macrocarpal E (**189**), macrocarpal Q (**190**), eucarobustol E (**191**), euglobal-V (**192**), euglobal-III (**193**), 1-(2,6-dihydroxy-4-methoxy-3,5-dimethylphenyl)-2-methylbutan-1-one (**194**), and 1-(2,4-dihydroxy-6-methoxy-3,5-dimethylphenyl)-3-methylbutan-1-one (**195**). The T-cell proliferation model was utilized to evaluate the immunosuppressive effects of compounds. Compounds **178**–**195** displayed moderate inhibitory activity, with IC_50_ values ranging from 10.2 to 132.9 μM [73].

Prenyllongnol A (**196**), prenyllongnol B (**197**), prenyllongnol C (**198**), and prenyllongnol D (**199**) were extracted from *Hypericum longistylum*. Compounds **196**–**199** demonstrated significant immunosuppressive effects on the proliferation of murine splenocytes induced by ConA, with IC_50_ values ranging from 2.98 to 6.34 μM [74].

The cultivated *Dendrobium devonianum* Paxt produced two bibenzyl-phenylpropane hybrids, named dendrophenene A and dendrophenene B (**200** and **201**). Bioassays conducted on mouse splenic lymphocytes stimulated with ConA and LPS revealed that compounds **200** and **201** inhibited the proliferation of T lymphocytes, with IC_50_ values of 0.17 μM and 2.47 μM, respectively. Additionally, they showed immunosuppressive effects on LPS-induced B-cell proliferation, with IC_50_ values ranging from 28.3 μM to 52.6 μM. DXMS was selected as the positive control against ConA-induced T-cells and LPS-induced B-cell proliferation, with IC_50_ values of 2.0 and 1.1 μM, respectively [75].

Tyrosol (**202**) and 3,4-dihydroxy-ethyl ester (**203**) were isolated from *Hydnora abyssinica* ethanolic extract, which revealed immunosuppressive activity against reactive oxygen species from MNCs. Compounds **202** and **203** exhibited inhibitory effects against superoxide production at concentrations of 100 μg/mL [76]. A phenolic compound called daldiniols A (**204**), derived from the endophytic fungus *Daldinia* sp. TJ403-LS1, was isolated from the medicinally valuable plant *Anoectochilus roxburghii*. Compound **204** exhibited remarkable immunosuppressive activity against the proliferation of murine splenocytes activated by LPS and anti-CD3/anti-CD28 mAbs, with an identical IC_50_ value of 0.06 μM [77]. 

Two phenolic glycosides, named 2-phenylpropanoate-2-*O*-*β*-D-apiofuranosyl-(1→6)-*O*-*β*-D–glucopyranoside (**205**) and 3,4,5-trimethoxyphenol-*β*-D-apiofuranosyl-(1→6)-*O*-*β*-D-glucopyranoside (**206**), were isolated from the barks of *Cinnamomum cassia*, which had an obvious immunosuppressive activity against T-cell proliferation with IC_50_ values of over 200 μM and 12.5 μM, respectively [72].

Four polyprenylated acylphloroglucinols, (+)-hyperzewalsin B (**207**), (−)-hyperzewalsin B (**208**), hyperzewalsin E (**209**), and lupulone D (**210**), were isolated from the aerial parts of *Hypericum przewalskii* Maxim. Compounds **207**–**210** were tested for their immunosuppressive activities in LPS-stimulated splenocytes, with IC_50_ values ranging from 6.61 to 7.36 μM [78]. Ten mycophenolic acid derivatives (**211**–**220**) were obtained from the fungus *Penicillium bialowiezense*. The name and immunosuppressive activities are shown in Table S6. Compounds **211**–**220** showed significant inhibitory potency, with IC_50_ values ranging from 3.27 to 24.68 μM [79].

Hyperformitin A (**221**), hyperformitin B (**222**), hyperformitin C (**223**), hyperformitin D (**224**), hyperformitin E (**225**), hyperformitin G (**226**), hyperformitin H (**227**), hyperformitin J (**228**), hyperformitin K (**229**), hyperformitin L (**230**), and hyperformitin M (**231**) were isolated from *Hypericum perforatum*. Compounds **221**–**231** were tested for their immunosuppressive activities against LPS-induced B lymphocyte proliferation, with IC_50_ values ranging from 4.1 to over 10 μM [80]. 

Two acylphloroglucinols, przewalcyrones C and D (**232** and **233**), were isolated from *Hypericum przewalskii* Maxim. Compounds **232** and **233** exhibited remarkable immunosuppressive activity against anti-CD3/anti-CD28 mAbs, activating murine splenocyte proliferation with IC_50_ values of 5.01 μM and 5.26 μM, respectively [81]. A previous polycyclic polyprenylated acylphloroglucinol, cumilcinol E (**234**), was isolated from *Hypericum wilsonii*. Compound **234** demonstrated significant inhibitory activity on ConA-induced T-lymphocyte proliferation, with an IC_50_ value of 4.80 μM [82]. 

### 3.4. Flavonoids

**Figure 9:**
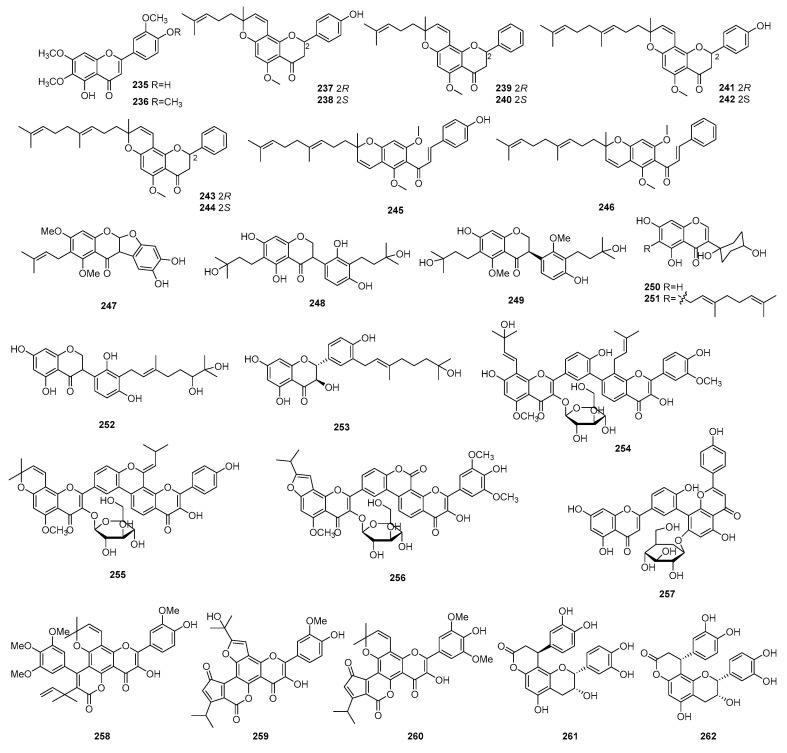
Structures of flavonoids **235**–**262**.

Some flavonoid compounds have shown immunosuppressive properties in the last decade. The structures, sources, selected models, and immunosuppressive activity are shown in Figure 9 and Table S7.

Two flavonoids named jaceosidin (**235**) and eupatilin (**236**) were isolated from *Artemisia argyi.* Compounds **235** and **236** showed inhibitory activity on LPS-stimulated BV-2 microglial cell proliferation with IC_50_ values of 1.9 and 4.0 μM [83]. Tsaokonol A (**237**), tsaokonol B (**238**), tsaokonol C (**239**), tsaokonol D (**240**), tsaokonol E (**241**), tsaokonol F (**242**), tsaokonol G (**243**), tsaokonol H (**244**), tsaokonol I (**245**), and tsaokonol J (**246**) were isolated from the fruits of *Amomum tsao-ko*. Compounds **237**–**246** were evaluated for their inhibitory effects on LPS-induced NO production, with IC_50_ values ranging from 10.6 to 41.5 μM [28].

Compounds **247**–**253** were extracted from *Campylotropis hirtella*, whose names are shown in Table S7. The immunosuppressive activity of compounds **247**–**253** was tested, which indicated these compounds had moderate activities against T-cell and B-cell proliferation with IC_50_ values ranging from 0.26 to 53.16 μM and 1.55 to 29.12 μM, respectively. CsA was selected as the positive control against T and B-cell proliferation, with IC_50_ values of 0.01 and 0.97 μM, respectively [84].

Four biflavonoid derivatives (**254**–**257**) were isolated from *Citrus medica* L. var. *sarcodactylis Swingle*. The names and immunosuppressive activities of other compounds are shown in Table S7. Compound **254**–**257** exhibited significant inhibitory activity against ConA-induced T-lymphocyte proliferation, with IC_50_ values ranging from 16.83 to 50.90 μM [85].

Five dimethylallylated flavonolignans (**258**–**262**) were isolated from the fruit of *Hippophae rhamnoides* L. The name and immunosuppressive activity are shown in Table S7.

Compounds **258**–**262** showed excellent inhibitory effects against ConA-induced T lymphocyte proliferation, with IC_50_ values ranging from 19.42 to 48.05 μM [86].

### 3.5. Steroids

**Figure 10:**
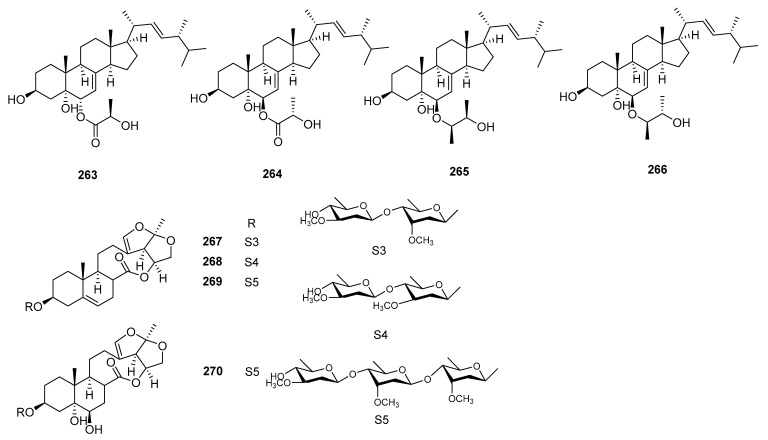
Structures of steroids **263**–**270**.

Some steroid compounds have shown immunosuppressive properties in the last decade. The structures, sources, selected models, and immunosuppressive activity are shown in Figure 10 and Table S8.

Quadristerols B, quadristerols C, quadristerols D, and quadristerols F (**263**–**266**), four ergosterols, were obtained from *Aspergillus quadrilineata*. Compounds **263** and **264** showed excellent inhibitory effects against ConA-induced T lymphocyte proliferation, with IC_50_ values of 7.43 and 3.95 μM, respectively. Compounds **265** and **266** strongly inhibited LPS-induced B lymphocyte proliferation, with IC_50_ values of 10.96 and 7.47 μM, respectively [87].

Six new C_21_ steroidal glycosides, named atratcynoside A (**267**), atratcynoside B (**268**), atratcynoside C (**269**), and datratcynoside E (**270**), were isolated from the root of *Cynanchum atratum*. Compounds **267**–**270** were subjected to immunosuppressive activities by an in vitro model of ConA-induced proliferation of T-lymphocytes. Compounds **267**–**270** exhibited significant dose-dependent immunosuppressive activities, with IC_50_ values ranging from 3.3 to 7.0 μM [88].

### 3.6. Others

**Figure 11:**
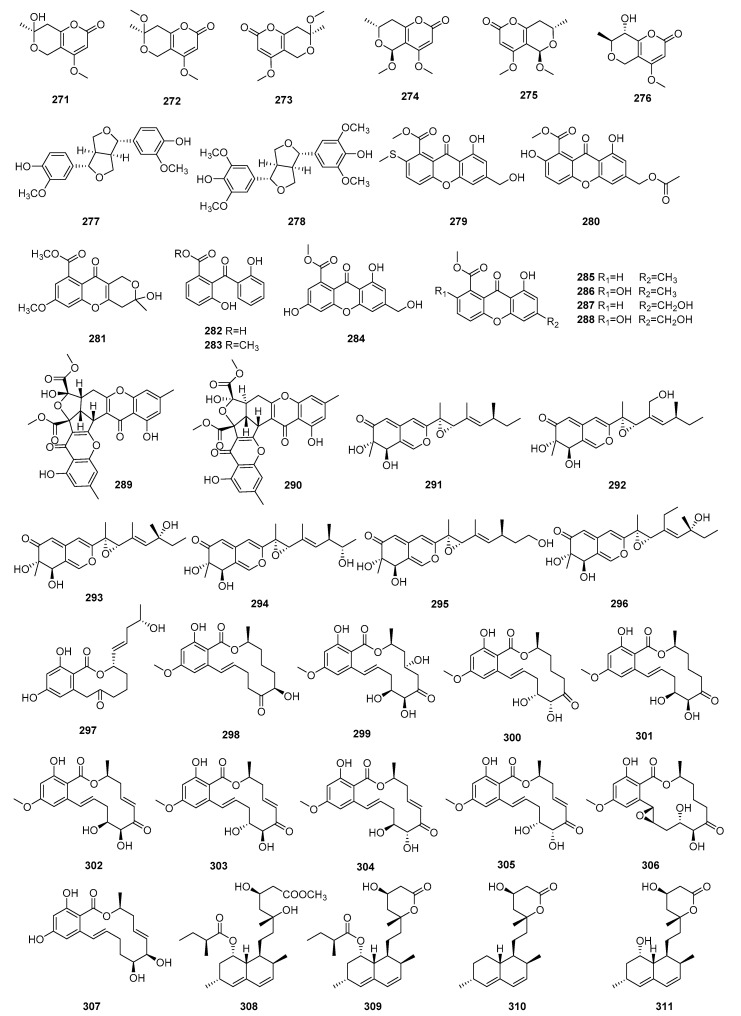
Structures of others **271**–**311**.

**Figure 12:**
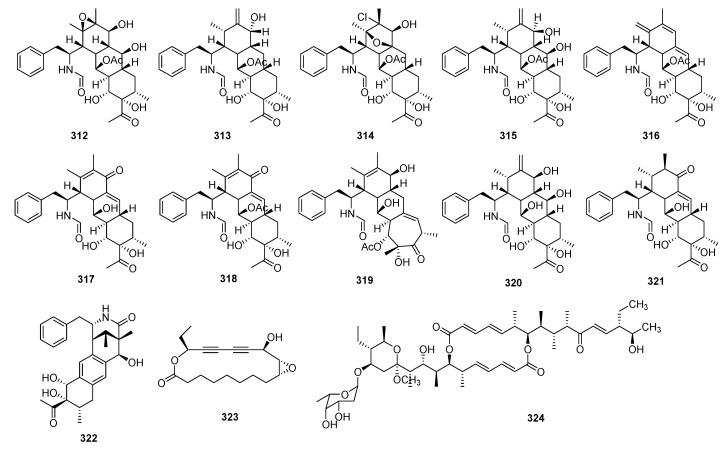
Structures of others **312**–**324**.

In addition, the structures, sources, selected models, and immunosuppressive activities of other compounds are shown in Figure 11 and Figure 12 and Table S9.

Six pyrone derivatives (+)-Adprepyrone B (**271**), (+)-Adprepyrone D (**272**), (−)-Adprepyrone D (**273**), (+)-Adprepyrone E (**274**), (−)-Adprepyrone E (**275**), 6-[(*E*)-3-Hydroxyprop-1-enyl]-4-methoxy-5-methyl-2-pyrone (**276**), were derived from the fungus *Talaromyces adpressus*. Compounds **271**–**276** demonstrated moderate inhibitory effects on ConA-induced proliferation of T lymphocytes, with IC_50_ values ranging from 8.9 to 19.8 μM [89]. Pinoresinol (**277**) and syringaresinol (**278**) had been isolated from the stems of *Epigynum cochinchinensis*. Compounds **277** and **278** inhibit ConA-stimulated proliferation of mouse splenocytes with IC_50_ values of 12.5 and over 50 μM, respectively [90].

Two xanthone derivatives, sydoxanthone B (**279**) and 13-*O*-acetylsydowinin B (**280**), were isolated from the fungus *Aspergillus sydowii*, occurring in the livewort *Scapania ciliata* S. Lac. Compound **279** inhibited T and B-cell proliferation with IC_50_ values of 22.53 and 12.3 μM, respectively. Meanwhile, compound **280** inhibited the LPS-induced B-cells with an IC_50_ value of 172.7 μM [91]. A chromone, diaporchromone A (**281**), was isolated from the culture of *Phomopsis asparagi* DHS-48. Compound **281** showed moderate to weak immunosuppressive activity against T and B lymphocyte cells with IC_50_ values of 34 μM and 117 μM, respectively [92].

Nine polyketides (**282**–**290**) were isolated from the leaves of *Sonneratia apetala*: Peniphenone (**282**), methyl peniphenone (**283**), conioxanthone A (**284**), methyl 8-hydroxy-6-methyl-9-oxo-9H-xanthene-1-carboxylate (**285**), pinselin (**286**), sydowinin B (**287**), sydowinin A (**288**), remisporine B (**289**), and epiremisporine B (**290**). The immunosuppressive activity of compounds **282**–**290** was tested, which indicated these compounds had moderate activities against T-cell and B-cell proliferation with IC_50_ values ranging from 5.9 to 30.8 μM and 7.1 to 32.4 μM, respectively [93].

Seven undescribed side chains containing azaphilones, pestaphilone A (**291**), pestaphilone B (**292**), pestaphilone C (**293**), pestaphilone D (**294**), pestaphilone E (**295**), and pestaphilone F (**296**), were isolated from the *Anoectochilus roxburghii* endophytic fungus *Pestalotiopsis oxyanthi*. In bioassay, compounds **291**–**296** displayed potential immunosuppressive activity in ConA-induced T lymphocyte proliferation, with IC_50_ values ranging from 9.36 to 35.21 μM, respectively [94].

Eleven hypothemycin-type resorcylic acid lactones (**297**–**307**) were obtained from the solid rice-based culture of *Podospora* sp. G214. The name and immunosuppressive activities are shown in Table S9. Compounds **297**–**307** exhibited potent immunosuppressive activities against ConA-induced T-cell proliferation with IC_50_ values ranging from 6.0 to 25.1 μM and LPS-induced B-cell proliferation with IC_50_ values ranging from 6.2 to 29.1 μM [95]. 

Four phenolics, named 1-naphthaleneheptanoic (**308**), monacolin K (**309**), monacolin L (**310**), and monacolin J (**311**), were isolated from the endophytic fungus *Aspergillus* sp. found in *Tripterygium wilfordii*. Compounds **308**–**311** showed potential immunosuppressive activity against anti-CD3/anti-CD28 mAbs-irritated murine splenocyte proliferation, with IC_50_ values ranging from 5.30 μM to 16.51 μM [96].

Compounds **312**–**322** were specifically immunosuppressive to T lymphocytes with IC_50_ values of 12.6 and 70.9 μM and LPS-induced B-cell proliferation with IC_50_ values ranging from 2.4 to 88 μM, respectively [75]. Ivorenolide B (**323**), a 17 membered macrolide featuring conjugated acetylenic bonds, was isolated from *Khaya ivorensis*. Compound **323** showed inhibitory activity on B lymphocyte proliferation with an IC_50_ value of 4.80 μM [97].

A new asymmetric macrodiolide immunosuppressant, named efophylin B (**324**), is from *Streptomyces malaysiensis* DSM 4137. Compound **324** demonstrated significant inhibitory activity against T lymphocyte proliferation, with an IC_50_ value of 24.6 μM [98].

## 4. Structure-Activity Relationship

In this review, the structure-activity relationship of terpenoids, alkaloids, phenolic compounds, flavonoids, and others with immunosuppressive activity was analyzed. Within these categories, variations in substituents, substitution sites, and configuration result in varying degrees of immunosuppressive activity.

Sesquiterpenoids **1**–**9** had a similar guaiacosane skeleton. Comparing the immunosuppressive activity in pairs, compounds **1**–**5** with C-8-Ac had strong activity for LPS-induced proliferation of B lymphocyte cells. It seems that C-8-Ac increased immunosuppressive activity. Compounds **47**–**51** possessed a similar farnesanes-type diterpenoid skeleton. Compound **49** exhibited the highest activity against T-cell and B-cell proliferation, possibly attributed to the varying oxidation levels of these compounds at C-4. This suggests that increasing the oxidation levels at C-4 may enhance antibacterial activity.

Triterpenoids **117** and **118**, with highly similar chemical structures, exhibited inhibitory activity on murine splenocyte proliferation, with **117** being more potent than **118**. The structural disparity lies in the oxidation of the C-3 hydroxyl group in **117**, which potentially impacts its activity.

In an immunosuppressive activity assay comparing alkaloids, both **173** and **174** exhibited inhibitory activity against LPS-induced B-cell proliferation, with compound **173** demonstrating greater potency than compound **174**. The structural distinction lies in the presence of a methoxy group at the C-6 position in compound **173**, which suggests that the methoxy group at the C-6 may increase the antibacterial activity.

The immunosuppressive activity assay of phloroglucinol derivatives was also performed.

Compounds **178** and **179**, possessing isopropyl functionality at C-4′, exhibited significant efficacy in promoting LPS-induced proliferation of ConA-induced murine T-cells. It seems that isopropyl functionality at C-4′ increases immunosuppressive activity.

## 5. Conclusions

**Figure 13:**
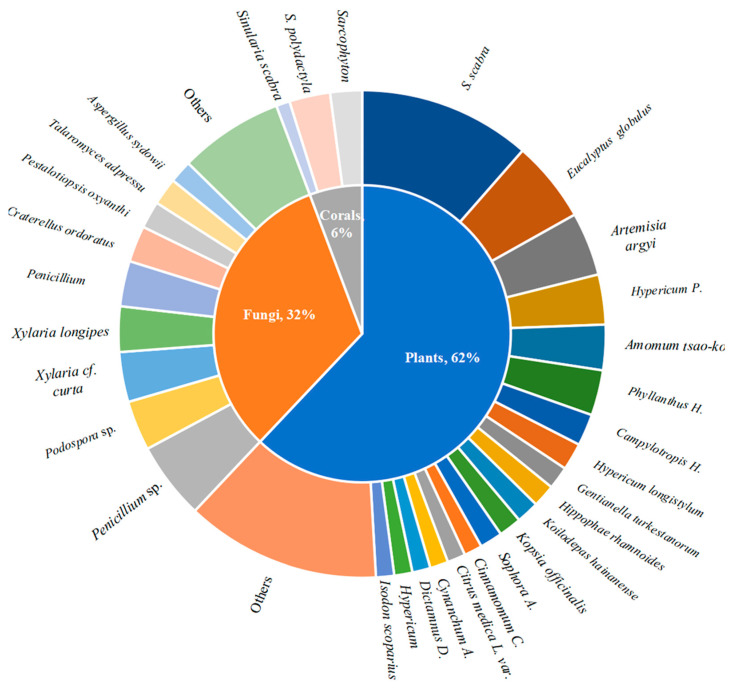
Immunosuppressive bioactive compounds sourced from natural products.

In this review article, we have provided information on the source, structure, and immunosuppressive activity of each discussed compound. A total of 324 small-molecule compounds with immunosuppressive activity have been discovered, and their sources are counted in Figure 13. This figure showed that small-molecule compounds with immunosuppressive activity have been isolated from natural sources, including plants (62%), animal corals (6%), and fungi (32%). Among these compounds, the main plant-derived molecules identified are sesquiterpene lactones in *A. argyi*, specifically compounds **1**–**6** and **9**–**13**. The primary coral-derived molecules identified are diterpenoids found in *S. scabra* and *S. polydactyla*. Additionally, marine coral-derived molecules such as sesquiterpenoids found in *C*. *odorants* (**29**–**36**) and diterpenoids in *X*. *longipes* (**52**–**59**) have been identified as significant contributors to this field of study. 

By comparing the monomer components summarized in this review, it can be found that phenols and terpenes have good immunosuppressive activity. In contrast, monomeric components, such as alkaloids and saponins, are less studied and deserve further exploration. A total of 57 terpenoids have been identified, which is the most abundant type of compound. CsA is an immunosuppressive agent that inhibits the proliferation of T and B lymphocytes. As a positive drug for immunosuppressive activity screening, its IC_50_ values in various studies ranged from 0.01 to 1 μM. By comparing positive drugs, it can be found that some compounds have great potential for development. Among them, compounds **49**, **151**, **173**, **200**, **204**, and **247** have demonstrated good immunosuppressive activity with IC_50_ values less than 1 μM, while one hundred and nine other compounds have IC_50_ values less than 10 μM. The foundation and core of innovative drugs is activity. These compounds may hold great potential to become new natural therapeutic agents for treating autoimmune diseases [99,100,101]. 

Compounds with good immunosuppressive activity have enormous potential as immunosuppressive agents. After discovering compounds with good immunosuppressive activity from natural products, the process of transforming them into candidate drugs should be optimized. This process includes system detection of its toxicity, solubility, and target, among others. It is necessary to conduct a systematic evaluation of the pharmacology, toxicology, safety, and efficacy of candidate drugs during the development process. The reviewed products in the article are all natural products. Due to the relatively low content of compounds and the difficulty of their synthesis, most of them only undergo simple activity screening. The immunosuppressive mechanisms of several compounds have been studied. 

Eucalyptin C (**180**) had been found to be not only an immunosuppressive agent but also a selective PI3K inhibitor. Compound **180** induces apoptosis and inhibits activation of primary splenic cells in mice with allergic contact dermatitis by inhibiting downstream expression of PI3K [73]. BC-1 (**121**) not only inhibited the proliferation of splenic lymphocytes and phagocytosis of macrophages but also significantly reduced Th1/Th2 cytokines (IL-2, IFN-*γ*) in CD4^+^ cells. With the production of IL-4 and IL-10, compound **121** can inhibit the activation of JNK, ERK, and PI3K/AKT signaling pathways. These results indicate that compound **121** has a potential downregulation effect on the immune system and may serve as an immunosuppressive agent for the treatment of CD4^+^ inflammation and adverse immune reactions [52]. Their research methods on the mechanism of action may have good reference significance for studying the mechanism of action of structurally similar compounds. 

However, there are limitations in studying monomeric fractions due to their small amounts obtained through complex extraction processes [102,103,104]. Solving extraction problems is crucial if we want these monomeric components to be used clinically [105,106]. Therefore, new methods are needed to accurately determine the composition of natural active substances as well as assess their effectiveness in human and animal models [107,108,109]. This review evaluates the immunosuppressive activity of different experiments using the main criterion, IC_50_. This method can compare the activity of different chemical structures and estimate their future potential. The objective issue currently being studied is the need for appropriate, high-throughput synthesis methods to prepare small molecules.

In terms of food and chemistry, many plants derived from the compounds reviewed in this article may also have immunosuppressive activity. Among them, plants with the same origin in food-medicine herbs developed into a series of foods. In agriculture, the agricultural planting industry of medicinal and edible plants can be extended and expanded while promoting economic development.

## Figures and Tables

**Table 1:** Establishment of models of the immunosuppressive efficacy in vitro.

Number	Model	Inducing Drugs
1	human peripheral blood T-lymphocytes	ConA
2	human mononuc-lear cell + stimulated RAW264.7 macrophage cell	ConA
3	isolated peritoneal macrophages	LPS
4	splenocytes	ConA/LPS
5	RAW264.7 macrophage cell	ConA
6	stimulated spleen cells	anti-CD3/anti-CD28
7	T-cell proliferation	phytohaemagglutinin
8	BV-2 microglia	LPS

**Table 2:** Establishment of models of the immunosuppressive efficacy in vivo.

Number	Model Making Drug	Animal	Mode of Administration
1	Mitogen and OVA	ICR mice	immunized subcutaneously in one hind limb of OVA and in saline soln
2	Cyclophosphamide	C57BL/6 mice	intraperitoneal injection
3	CCl_4_	Wistar rats	intraperitoneal injection
4	Xylene	Balb/c mice	injected xylene into the left ear of each mouse
5	DTH	Balb/c mice	smearing DNCB on the shaved abdominal skin of the mice
6	SRBC	Swiss albino mice	the mice were primed with SRBC

## Data Availability

This article is a review article without any research data. All analysis results have been presented in the manuscript.

## Supplementary Materials

The following supporting information can be downloaded at: https://www.mdpi.com/article/10.3390/molecules29102359/s1. Tables S1–S9: Compounds **1**–**324** with Immunosuppressive Effects. References [28,29,30,31,32,33,34,35,36,37,38,39,40,41,42,43,44,45,46,47,48,49,50,51,52,53,54,55,56,57,58,60,61,62,63,64,65,66,67,68,72,73,74,75,76,77,78,79,80,81,82,83,84,85,86,87,88,89,90,91,92,93,94,95,96,97,98] are cited in the Supplementary Materials.
