# Supplementary material for: Research Progress of Natural Active Substances with Immunosuppressive Activity
Source: Molecules. 2024 May 16;29(10):2359. doi: 10.3390/molecules29102359 (PMC11124018; doi:10.3390/molecules29102359)
Supplement: Supplementary file 1 [file molecules-29-02359-s001.zip › molecules-2990204-supplementary.pdf]

Supplementary Materials for

Research Progress of Natural Active Substances with Immunosuppressive Activity

Fei Shao [1], Qiying Shen [1], Zhengfei Yang [3], Wenqian Yang [1], Zixiang Lu [1], Jie Zheng [1],

Liming Zhang [1,*] and Hangying Li [1,2,*]

[1] School of Pharmacy, Ningxia Medical University, Yinchuan 750004, China

[2] Key Laboratory of Craniocerebral Diseases of Ningxia Medical University,Yinchuan 750004, China

[3] School of Traditional Chinese Medicine, Ningxia Medical University,Yinchuan 750004, China

[*] Correspondence: nyzlm@163.com (L.Z.); lihy17@lzu.edu.cn (H.L.)

List of Supplementary Materials

Table S1. Compounds **1**–**36** with Immunosuppressive Effects........3

Table S2. Compounds **37**–**115** with Immunosuppressive Effects........6

Table S3. Compounds **116**–**136** with Immunosuppressive Effects........10

Table S4. Compounds **137**–**149** with Immunosuppressive Effects........12

Table S5. Compounds **150**–**176** with Immunosuppressive Effects........13

Table S6. Compounds **177**–**234** with Immunosuppressive Effects........15

Table S7. Compounds **235**–**262** with Immunosuppressive Effects........19

Table S8. Compounds **263**–**270** with Immunosuppressive Effects........22

Table S9. Compounds **271**–**324** with Immunosuppressive Effects........23

Table S1. Compounds 1–36 with Immunosuppressive Effects.

Noumber	Compounds	Type	Source	Model 1	Activities IC_{50}	Model 2	Activities IC_{50}	Reference
1	Argyinolide G	Plant	*Artemisia argyi*	LPS stimulated in BV-2 microglial cells	5.3 μM	/	/	[28]
2	8α-acetoxy-3β-chloro-1α,4α-dihydroxyguai-9,11(13)-dien-6α,12-olide				3.2 μM		/	
3	8α-acetoxy-3α-chloro-1α,4β-dihydroxyguai-9,11(13)-dien-6α,12-olide				6.9 μM		/	
4	8α-acetoxy-3β-chloro-1α,4α-dihydroxyguai-10(14),11(13)-dien-6α,12-olide				4.2 μM		/	
5	8α-acetoxy-3α-chloro-1β,2β-epoxy-4β,10α-dihydroxy-5α,7αH-guai-11(13)-en-12,6α-olide				22.2 μM		/	
6	3β-chloro-1α,2α-epoxy-4α,10α-dihydroxy-5α,7αH-guai-11(13)-en-12,6α-olide				6.4 μM		/	
7	Tremutin A	Fungi	*Irpex lacteus*	ConA-induced T-cell proliferation	/	LPS-induced B-cells proliferation	22.4 μM	[29]
8	Tremutin B				16.7 μM		13.6 μM	

9	Canin	Plant	*A. argyi*	anti-CD3/anti-CD28 stimulated spleen cells	2.7 μM	/	/	[28]
10	*seco*-tanapartholide B				1.0 μM	/	/	
11	*seco*-tanapartholide A				1.2 μM	/	/	
12	Arteglasin A				1.9 μM	/	/	
13	8-acetylarteminolide				3.2 μM	/	/	
14	Maydispenoid A	Fungi	*Bipolaris maydis*	anti-CD3/anti-CD28 stimulated spleen cells	5.28 μM	/	/	[30]
15	Maydispenoid B				9.38 μM		/	
16	Antroxazole A	Fungi	*Antrodiella albocinnamomea*	/	/	LPS-induced B-cells proliferation	16.3 μM	[14]
17	(+)-aspersydowin A	Fungi	*Aspergillus sydowii*	anti-CD3/anti-CD28 stimulated spleen cells	>40 μM	LPS-induced B-cells proliferation	10.9 μM	[31]
18	(+)-aspersydowin B				>40 μM		17.6 μM	
19	(7*S*)-(+)-7-*O*-methylsydono				>40 μM		13.4 μM	
20	Parasubolide D	Plant	*Parasenecio albus*	LPS-induced spleen-cell proliferation	23.1 μM	/	/	[32]
21	Parasubolide E				33.8 μM	/	/	
22	Parasubolide L				26.6 μM	/	/	
23	Steccherin A	Fungi	*Steccherinum ochraceum*	ConA-induced T-cell proliferation	>40 μM	LPS-induced B-cells proliferation	26.3 μM	[33]
24	Steccherin B				>40 μM		>40 μM	
25	Steccherin C				37.8 μM		6.2 μM	
26	Steccherin D				>40 μM		16.1 μM	
27	Soltorvum A	Plant	*Solanum torvum.*	ConA-induced T-cell proliferation	27.0 μM	/	/	[34]
28	Soltorvum B				18.0 μM	/	/	

29	Craterodoratin C	Fungi	*Craterellus ordoratus*	ConA-induced T-cell proliferation	/	LPS-induced B-cells proliferation	12.62 μM	[35]
30	Craterodoratin J				/		19.4 μM	
31	Craterodoratin L				/		13.7 μM	
32	Craterodoratin M				/		15.4 μM	
33	Craterodoratin N				/		13.2 μM	
34	Craterodoratin O				/		17.1 μM	
35	Craterodoratin Q				31.5 μM		/	
36	Craterodoratin S				/		22.6 μM	

Table S2. Compounds 37–115 with Immunosuppressive Effects.

Noumber	Compounds	Type	Source	Model 1	Activities IC_{50}	Model 2	Activities IC_{50}	Reference
37	Ineleganolide	Coral	*Sinularia scabra* and *Sinularia polydactyla*	ConA-induced T-cell proliferation	>50 μM	LPS-induced B-cells proliferation	>50 μM	[36]
38	Yonarolide				>50 μM		>50 μM	
39	Scabrolide A				>50 μM		>50 μM	
40	Cinncassiol G	Plant	*Cinnamomum cassia*	ConA-induced T-cell proliferation	/	/	/	[37]
41	Cinnacasol				/	/	/	
42	3,5,10-O triacetyl-8-*O*-isobutanoyl-14-*O*-benzoylcyclomyrsinol	Plant	*Euphorbia kopetdaghi* Prokh	PHA-activated T-Cell proliferation	1.83 mg /mL	/	/	[38]
43	Xylarilongipin A	Fungi	*Xylaria longipes* HFG1018	ConA-induced T-cell proliferation	22.4 μM	LPS-induced B-cells proliferation	22.4 μM	[39]
44	Tripterifordin	Plant	*Ligularia fischeri*	human B lymphoblast HMy2.CIR cells	56.3 μM	/	/	[40]
45	16α,17-dihydroxy-ent-kaur-20-al.				13.3 μM	/	/	
46	ent-2β-hydroxymanool				31.4 μM	/	/	
47	Koilodenoid D	Plant	*Koilodepas hainanense*	ConA-induced T-cell proliferation	23.8 μM	LPS-induced B-cells proliferation	10.2 μM	[41]
48	Koilodenoid G				3.1 μM		2.2 μM	
49	ent-5α,2,15-dioxodolabr-3-ene-3,16-diol				0.8 μM		0.3 μM	
50	ent-5α,3,15-dioxodolabr-1,4(18)-diene-2,16-diol				30.5 μM		4.7 μM	
51	ent-16-nor-5α,13α(methyl)-2-oxodolabra-3-en-3-ol-15-oicacid				3.4 μM		3.1 μM	

52	Xylarinorditerpene B	Fungi	*Xylaria longipes* HFG1018	ConA-induced T-cell proliferation	11.1 μM	LPS-induced B-cells proliferation	35.1 μM	[42]
53	Xylarinorditerpene C				6.6 μM		38.2 μM	
54	Xylarinorditerpene D				21.1 μM		51.8 μM	
55	Xylarinorditerpene E				14.9 μM		49.4 μM	
56	Xylarinorditerpene I				1.0 μM		16.1 μM	
57	Xylarinorditerpene N				4.0 μM		46 μM	
58	14α,16-epoxy-18-norisopimar-7-en-4α-ol				27.2 μM		40 μM	
59	Agatadiol				15.6 μM		47.2 μM	
60	Robustaditerpenes C	Fungi	*Ilyonectria robusta*	ConA-induced T-cell proliferation	/	LPS-induced B-cells proliferation	17.42 μM	[43]
61	Robustaditerpenes E				75.22 μM		/	
62	Scopariusicide I	Plant	*Isodon scoparius*	ConA-induced T-cell proliferation	10.1μM	/	/	[44]
63	Scopariusicide J				9.4μM	/	/	
64	Scopariusicide L				16.3μM	/	/	
65	Scopariusic Acid	Plant	*Isodon scoparius*	ConA-induced T-cell proliferation	2.6 μM	/	/	[46]
66	Ceforloid F	Plant	*Cephalotaxus fortunei var. alpina and C. sinensis*	ConA-induced T-cell proliferation	1.93 μM	/	/	[45]
67	Xiguscabrolide H	Coral	*S. scabra* and *S. polydactyla*	ConA-induced T-cell proliferation	45.7 μM	LPS-induced B-cells proliferation	44.1 μM	[36]
68	10-epi-gyrosanolide E				>50 μM		>50 μM	
69	5-Epi-sinuleptolide				39.6 μM		>50 μM	
70	Norcembrene 5				>50 μM		>50 μM	
71	Scabrolide D				>50 μM		>50 μM	

72	Scabrolide G	Coral	*scabra* and *S. polydactyla*	ConA-induced T-cell proliferation	23.7 μM	LPS-induced B-cells proliferation	20.5 μM	[36]
73	Sinularcasbane O				>50 μM		>50 μM	
74	Gyrosanolide F				29.1 μM		>100μM	
75	Sinuleptolide				8.5 μM		21.7 μM	
76	Xiguscabrate A	Plant	*S. scabra*	ConA-induced T-cell proliferation	>50 μM	LPS-induced B-cell proliferation	46.5 μM	[47]
77	Xiguscabrate B				8.4 μM		25.6 μM	
78	Xiguscabral A				15.8 μM		44 μM	
79	Xiguscabrol A				5.5 μM		>50 μM	
80	Xiguscabrol B				3.9 μM		44.8 μM	
81	8-*epi*-xiguscabrol B				2.3 μM		34.9 μM	
82	(1*Z*,5*E*,7*E*,11*E*)-8-isopropyl-5,11-dimethylcyclo tetradeca-				>50 μM		>50 μM	
83	11S,12S-epoxy-11,12-dihydrocembrene-C				39.2 μM		14 μM	
84	Sinulariol C				4.5 μM		46.8 μM	
85	Cembrene-A				44.3 μM		16.7 μM	
86	Pentaene-cembrene				>50 μM		39.3 μM	
87	(1*S*)-isoscarcophytol-A				27.1 μM		44.1 μM	
88	(1*E*,3*E*,7*E*,11*E*)-(l4*R*)-cembra-1,3,7,11-tetraen-14-ol				19.6 μM		>50 μM	
89	(1*S*,2*E*, 4*S*,7 *E*,11*E*) -2,7,11-cembratriene-4-ol				>50 μM		49.8 μM	
90	Nephthenol				10.7 μM		38.6 μM	
91	(3*E*,11*E*)-cembra-3,8(19),11,15-tetraene-7-ol				48.9 μM		42.5 μM	
92	Sarcophytol T				40.4 μM		32.1 μM	
93	Gibberosene B				33.5 μM		>50 μM	

94	(1*E*,3*E*,7*E*,11*R**12*R**)-15-(acetoxymethyl)cembra-11,12-epoxy-1,3,7-triene	Plant	*S. Scabra*	ConA-induced T-cell proliferation	>50 μM	LPS-induced B-cell proliferation	10.3 μM	[47]
95	(1*R*,3*S*,4*S*,7*E*,ll*E*)-3,4-epoxycembra-7,11,15-trie				>50 μM		>50 μM	
96	Lobocrassin C				23.1 μM		>50 μM	
97	Pseudoplexauric acid methyl ester				>50 μM		>50 μM	
98	(1*E*,3*E*,7*R*,8*R*,11*E*,l4*R*)-3,4-epoxy-14-acetatecembra-1,3,11-trien-14-ol				11.9 μM		40.1 μM	
99	(2*R*,11*S*,12*S*)-isosarcophytoxide				>50 μM		4.4 μM	
100	Sarcophytoxide				>50 μM		>50 μM	
101	Isosarcophine				47.8 μM		>50 μM	
102	(+)-marasol				27.4 μM		>50 μM	
103	Sarcophytol W				>50 μM		>50 μM	
104	Sinulariol Z				>50 μM		>50 μM	
105	Sinulariol Z				6.1 μM		52.9 μM	
106	Sinuflexibilin D				39.4 μM		34.5 μM	
107	Sinulariolide				19.5 μM		9.2 μM	
108	Sinuladiterpene G				>50 μM		>50 μM	
109	Sarcomililate 1a	Coral	the Hainan Soft Coral *Sarcophyton mililatensis*	ConA-induced T-cell proliferation	49.8 μM	LPS-induced B-cell proliferation	20.2 μM	[48]
110	Sarcomililate 1b				38.9 μM		22.1 μM	
111	Sarcomililatol B				>50 μM		>50 μM	
112	Sarcomililatols 2a				44.5 μM		18.7 μM	
113	Sarcomililatols 3a				>50 μM		49.5 μM	
114	Yalongene A				>50 μM		4.8 μM	
115	Sarcophytol M				11.4 μM		4.9 μM	

Table S3. Compounds 116–136 with Immunosuppressive Effects.

Noumber	Compounds	Type	Source	Model 1	Activities IC50	Model 2	Activities IC50	Reference
116	Munronoid P	Plant	*Munronia pinnata*	ConA-induced T-cell proliferation	2.73 μM	LPS-induced B-cell proliferation	34.88 μM	[49]
117	Triterhyper A	Plant	*Hypericum longistylum*	anti-CD3/anti-CD28 stimulated spleen cells	4.5 μM	/	/	[50]
118	Lupeol				18.3 μM		/	
119	3b-hydroxyoleane-6b-oxethyl-12-en-28-oicacid-3-*O*-b-D-glucuronic	Plant	*Epigynum griffithianum*	proliferation of mice splenocyte	2.5 μM	/	/	[14]
120	Schincarin C	Plant	*Schisandra*	LPS-induced B-cell proliferation	10.2 μM	/	/	[51]
121	BC-1	Plant	*Beesia calthaefolia*	anti-CD3/anti-CD28 stimulated spleen cells	9.5 μM	/	/	[52]
122	Dictabretol A	Plant	*Dictamnus dasycarpus*	ConA-induced T-cell proliferation	1.5 μM	/	/	[53]
123	Dictabretol B				＞20 μM		/	
124	Dictabretol C				1.8 μM		/	
125	Dictabretol D				1.5 μM		/	
126	Schincalactones A	Plant	*Schisandra*	LPS-induced B-cell proliferation	36.8 μM	/	/	[54]
127	Phainanolide A	Plant	*Phyllanthus hainanensis*	ConA-induced T-cell proliferation	364.75 μM	LPS-induced B-cell proliferation	245.47 μM	[55]
128	Phainanoid G				566.83 μM		456.63 μM	
129	Phainanoid H				16.15 μM		8.24 μM	
130	Phainanoid I				218.14 μM		305.38 μM	

131	Phainanoid A	Plant	*Phylanthus hainanensis*	ConA-induced T-cell proliferation	184.9 μM	LPS-induced B-cell proliferation	122.28 μM	[56]
132	Phainanoid B				192.8 μM		249.49 μM	
133	Phainanoid C				6.24 μM		2.35 μM	
134	Phainanoid D				8.28 μM		17.04 μM	
135	Phainanoid E				43.26 μM		4.38 μM	
136	Phainanoid F				2.04 μM		＜1.60 μM	

Table S4. Compounds 137–149 with Immunosuppressive Effects.

Noumber	Compounds	Type	Source	Model 1	Activities IC_{50}	Model 2	Activities IC_{50}	Reference
137	Peniandranoid A	Fungi	*Penicillium* sp	ConA-induced T cell proliferation(EC_{50})	19 μM	LPS-induced B-cells proliferation(EC_{50})	/	[56]
138	Peniandranoid B				/		50 μM	
139	Peniandranoid C				4.3 μM		11 μM	
140	Peniandranoid D				11 μM		8.8 μM	
141	Peniandranoid E				27 μM		12 μM	
142	Isopenicin C				23 μM		23 μM	
143	Isopenicin A				8.5 μM		8.5 μM	
144	Isopenicin B				23 μM		7.5 μM	
145	Nitidasin	Plant	*Gentianella turkestanorum*	anti-CD3/anti-CD28 stimulated spleen cells	12.31 μM	IFN-γ production	16.5 μM	[57]
146	Gentianelloid F				13.68 μM		14.66 μM	
147	Alborosin				14.31 μM		12.4 μM	
148	Colquhounoid D	Plant	Colquhounia coccinea var. mollis	induced by anti-CD3/CD4 monoclonal antibodies	8.38 μM	/	/	[58]
149	14-*epi*-colquhounoid D				5.79 μM	/	/	

Table S5. Compounds 150–176 with Immunosuppressive Effects.

Noumber	Compounds	Type	Source	Model 1	Activities IC_{50}	Model 2	Activities IC_{50}	Reference
150	Wilfordatin E	Plant	*Tripterygium wilfordii* Hook. f.	HEK293 cells induced by LPS	8.75 μM	/	/	[60]
151	Tripfordine A				0.74 μM		/	
152	Wilforine				15.66 μM		/	
153	Alopecine A	Plant	*Sophora alopecuroides*	ConA-induced T cell proliferation	>100 μM	LPS-induced B-cells proliferation	69.6 μM	[61]
154	Alopecine B				>100 μM		96.8 μM	
155	Alopecine C				>100 μM		59.5 μM	
156	Alopecine D				3.9 μM		3.7 μM	
157	Alopecine E				58.7 μM		9.4 μM	
158	Albifpyrrol B	Fungi	*Albifmbria viridis*	ConA-induced T-cell proliferation	/	LPS-induced B-cell proliferation	16.16 μM	[62]
159	Fumiquinazoline J	Fungi	*Aspergillus fumigatus* HQD24.	ConA-induced T-cell proliferation	29.38 μM	LPS-induced B-cell proliferation	162.58 μM	[63]
160	Fumigaclavine C				52.13 μM		/	
161	12-Methoxychanofruticosinic acid	Plant	*Kopsia officinalis*	human T cell proliferation (PBMCs)	27.8 μM	/	/	[64]
162	N(4)-methylkopsininate				21.6 μM			
163	Demethoxycarbonylkopsin				25.4 μM			
164	Rhazinilam				1.0 μM			

165	Gentianelloid A	Plant	*Gentianella turkestanorum*	anti-CD3/CD28 mAbs induced murine T lymphocytes proliferation	5.64 μM	/	/	[65]
166	Gentianelloid B				3.93 μM		/	
167	Eurysoloids A	Plant	Eurysolen gracilis	anti-CD3/CD28 mAbs induced murine T lymphocytes proliferation	17.4 μM	/	/	[66]
168	Eurysoloids B				15.94 μM		/	
169	Rhazinilam	Plant	*Pausinystalia yohimbe*	ConA-induced T-cell proliferation	16.8 μM	LPS-induced B-cell proliferation	13.5 μM	[65]
170	Ophiorrhine C	Plant	*Ophiorrhiza cantoniensis Hace*	ConA-induced T cell proliferation	23.6 μM	LPS-induced B-cell proliferation	8.7 μM	[66]
171	Ophiorrhine D				17.9 μM		68.5 μM	
172	Ophiorrhine E				＞200 μM		116.2 μM	
173	Ophiorrhine F	Plant	*Ophiorrhiza japonica*	ConA-induced T cell proliferation	＞200 μM	LPS-induced B-cells proliferation	＜0.4 μM	[67]
174	Ophiorrhine G				＞201 μM		47.3 μM	
175	Reserpine	Plant	*Rauvolfifia yunnanensis Tsiang*	human T cell	5.0 μM	/	/	[68]
176	11-hydroxyburnamine				5.9 μM			

Table S6. Compounds 177–234with Immunosuppressive Effects.

Noumber	Compounds	Type	Source	Model 1	Activities IC_{50}	Model 2	Activities IC_{50}	Reference
177	Hypaluton A	Plant	*Hypericum patulum*	LPS-induced B-cell proliferation	6.8 μM	/	/	[72]
178	Eucalyptin A	Plant	*Eucalyptus globulus.*	ConA-induced T-cell proliferation	18.2 μM	/	/	[73]
179	Eucalyptin B				19.1 μM		/	
180	Eucalyptin C				11.8 μM		/	
181	Eucalyptin D				10.2 μM		/	
182	Eucalyptin E				35.8 μM		/	
183	Eucalyptin F				26.3 μM		/	
184	Eucalyptin G				35.3 μM		/	
185	Macrocarpal A				20.4 μM		/	
186	Macrocarpal B				31.2 μM		/	
187	Macrocarpal C				69.6 μM		/	
188	Macrocarpal D				41.2 μM		/	
189	Macrocarpal E				42.4 μM		/	
190	Macrocarpal Q				52.3 μM		/	
191	Eucarobustol E				68.4 μM		/	
192	Euglobal-V				69.3 μM		/	
193	Euglobal-III				129.3 μM		/	
194	1-(2,6-dihydroxy-4-methoxy-3,5 -dimethylphenyl)-2-methylbutan -1-one				70.2 μM		/	

195	1-(2,4-dihydroxy-6-methoxy-3,5-dimethylphenyl)-3-methylbutan-1-one	Plant	*Eucalyptus globulus.*	ConA-induced T-cell proliferation	132.9 μM	/	/	[72]
196	Prenyllongnol A	Plant	*Hypericum longistylum*	ConA-induced T cell proliferation	3.44 μM	/	/	[74]
197	Prenyllongnol B				2.98 μM		/	
198	Prenyllongnol C				5.31 μM		/	
199	Prenyllongnol D				6.34 μM		/	
200	Dendrophenene A	Plant	*Dendrobium devonianum*	ConA-induced T cell proliferation	0.17 μM	LPS-induced B cell proliferation	28.3 μM	[75]
201	Dendrophenene B				2.47 μM		52.6 μM	
202	Tyrosol	Plant	*H. abyssinica*	PMNs and MNCs	/	/	/	[76]
203	3,4-dihydroxy-ethyl ester				/	/	/	
204	Daldiniols A	Plant	*Anoectochilus roxburghii*	LPS-induced B-cell proliferation	0.06 μM	/	/	[77]
205	2-phenylpropanoate-2-*O*-*β*-D-apiofuranosyl-(1→6)-*O*-*β*-D–glucopyranoside	Plant	*Cinnamomum cassia*	ConA-induced murine T cells	＞200 μM	/	/	[72]
206	3,4,5-trimethoxyphenol-*β*-D-apiofuranosyl-(1→6)-*O*-*β*-D-glucopyranoside				12.5 μM		/	
207	(＋)-Hyperzewalsin B	Plant	*hypericum przewalskii maxim*	LPS-induced B-cell proliferation	7.36 μM	/	/	[78]
208	(–)-hyperzewalsin B				7.14 μM		/	
209	Hyperzewalsin E				7.2 μM		/	
210	Lupulone D				6.61 μM		/	

211	8-*O*-methyl mycophenolic acid	Fungi	*Penicillium bialowiezense*	/	/	Mouse Splenocyte Proliferation	4.21 μM	[79]
212	3-hydroxy mycophenolic acid				/		1.23 μM	
213	6-(5-carboxy-3-methylpent-2-enyl)-7-hydroxy-3,5-dimethoxy-4-methylphthalan-1-one				/		2.76 μM	
214	6-(5-methoxycarbonyl-3-methylpent-2-enyl)-3,7-dihydroxy-5-methoxy-4-methylphthalan-1-one				/		9.12 μM	
215	6-(3-carboxybutyl)-7-hydroxy-5-methoxy-4-methylphthalan-1-one				/		>40 μM	
216	6-[5-(2,3-dihydroxy-1-carboxyglyceride)-3-methylpent-2-enyl]-7-hydroxy-5-methoxy-4 methylphthalan-1-one				/		19.65 μM	
217	6-[5-(1-carboxy-4-N-carboxylate)-3-methylpent-2-enyl]-7-hydroxy-5-methoxy-4-methylphthalan-1-one	Fungi	*Penicillium bialowiezense*	/	/	Mouse Splenocyte Proliferation	24.58 μM	
218	N-mycophenoyl-L-valine				/		30.56 μM	
219	N-mycophenoyl-L-phenyloalanine				/		>40 μM	
220	N-mycophenoyl-L-alanine				/		>40 μM	

221	Hyperformitin A	Plant	*Hypericum perforatum*	LPS-Induced Murine B Cell Proliferation	9.7 μM	/	/	[80]
222	Hyperformitin B				>10 μM		/	
223	Hyperformitin C				4.3 μM		/	
224	Hyperformitin D				9.3 μM		/	
225	Hyperformitin E				4.1 μM		/	
226	Hyperformitin G				9.2 μM		/	
227	Hyperformitin H				>10 μM		/	
228	Hyperformitin J				>10 μM		/	
229	Hyperformitin K				8.8 μM		/	
230	Hyperformitin L				>10 μM		/	
231	Hyperformitin M				>10 μM		/	
232	Przewalcyrone C	Plant	*Hypericum przewalskii* Maxim	anti-CD3/anti-CD28 stimulated spleen cells	5.01 μM	/	/	[81]
233	Przewalcyrone D				5.26 μM		/	
234	Cumilcinol E	Plant	*Hypericum wilsonii*	ConA-induced T-cell proliferation	4.803 μM	/	/	[82]

Table S7. Compounds 235–262 with Immunosuppressive Effects.

Noumber	Compounds	Type	Source	Model 1	Activities IC_{50}	Model 2	Activities IC_{50}	Reference
235	Jaceosidin	Plant	*Artemisia argyi*	LPS stimulated in BV-2 microglial cells	1.9 μM	/	/	[83]
236	Eupatilin				4 μM	/	/	
237	Tsaokonol A	Plant	*Amomum tsao-ko*	against LPS-induced RAW 264.7 macrophages	16.2 μM	/	/	[28]
238	Tsaokonol B				14 μM		/	
239	Tsaokonol C				10.6 μM		/	
240	Tsaokonol D				13.5 μM		/	
241	Tsaokonol E				41.5 μM		/	
242	Tsaokonol F				39.2 μM		/	
243	Tsaokonol G				26.1 μM		/	
244	Tsaokonol H				28.7 μM		/	
245	Tsaokonol I				30.6 μM		/	
246	Tsaokonol J				13.5 μM		/	
247	4′,5′-dihydroxy-5,7-dimethoxy-6-(3-methylbut-2-enyl)-coumaronochromone	Plant	*Campylotropis hirtella*	ConA-induced T-cell proliferation	0.28 μM	LPS-induced B-cell proliferation	1.55 μM	[84]
248	6,3′-di(3-hydroxy-3-methylbutyl)-5,7,2′ 4′-tetrahydroxyisoflavanone				79.13 μM		29.12 μM	
249	3(*R*)-6,3′-di(3-hydroxy-3-methylbutyl)-2′-methoxyl-5,7,4′-trihydroxyisoflavanone				18.12 μM		6.66 μM	
250	Hydroisoflavone C				53.16 μM		25.76 μM	

251	3-(1′,4′-dihydroxycyclohexyl)-6-geranyl-5,7,-dihydroxyisoflavone	Plant	*Campylotropis hirtella*	ConA-induced T-cell proliferation	3.25 μM	LPS-induced B-cell proliferation	2.68 μM	[84]
252	5,7,4′-trihydroxy-3′-[6,7-dihydroxy-3,7-dimethyl-2(*E*)-octenyl] isoflavone				4.52 μM		2.38 μM	
253	2R,3R-3′-[7-hydroxy-3,7-dimethyl-2(*E*)-octenyl]-2,3-trans-5,7,4′-trihydroxy-flavonol				4.34 μM		4.82 μM	
254	3‴,4′,4⁗,7-tetrahydroxy-3⁗,5-dimethoxy-3-*O*-*β*-D-glucopyranosyl-8-isopentenol-8‴-prenyl-(3′→7″)-biflavonoid	Plant	*Citrus medica L. var.*	ConA-induced T-cell proliferation	16.83 μM	/	/	[85]
255	3‴,4⁗-dihydroxy-5-methoxy-3-*O*-*β*-D-glucopyranosyl-7,8-(12,13-dimethylchromene)-9‴-isopropyl-(3′→7″)-biflavonoid				18.95 μM		/	
256	3‴,4⁗-dihydroxy-3⁗,5,5⁗-trimethoxy-3-*O*-*β*-D-glucopyranosyl-7,8-isopropylpentenone-9‴-carbonyl-(3′→7″)-biflavonoid				20.28 μM		/	
257	Amentoflavone 7″-*O*-*β*-D-glucopyranoside				50.90 μM		/	

258	2-(4'-hydroxy-3'-methoxyphenyl)-3-hydroxy-12,13-dimethylchromene-3",4",5"-12 9-trimethoxyphenyl-8"-(4"',5"'-dimethylallyl)-dipyrano-xanthene-4,9"-dione	Plant	*Hippophae rhamnoides* L.	ConA-induced T-cell proliferation	41.82 μM	/	/	[86]
259	2-(4'-hydroxy-3'-methoxyphenyl)-3-hydroxy-7,8-furan-10-isopropanol-1",5"-144 isopropylpentenone-pyranochromene-2",4,6"-trione				19.42 μM		/	
260	2-(4'-hydroxy-3',5'-dimethoxyphenyl)-3-hydroxy-12,13-dimethylchromene-149 1",5"-isopropylpentenone-pyranochromene-2",4,6"-trione				20.19 μM		/	
261	Cinchonain A				48.05 μM		/	
262	Cinchonain B				46.91 μM		/	

Table S8. Compounds 263–270 with Immunosuppressive Effects.

Noumber	Compounds	Type	Source	Model 1	Activities IC_{50}	Model 2	Activities IC_{50}	Reference
263	Quadristerol B	Fungi	*Aspergillus quadrilineata*	ConA-induced T-cell proliferation	7.43 μM	LPS-induced B-cell proliferation	/	[87]
264	Quadristerol C				3.95 μM		/	
265	Quadristerol D				/		10.96 μM	
266	Quadristerol E				/		7.47 μM	
267	Atratcynoside A	Plant	*Cynanchum atratum*	ConA-induced T-cell proliferation	3.3 μM	/	/	[88]
268	Atratcynoside B				7 μM		/	
269	Atratcynoside C				6.7 μM		/	
270	Atratcynoside E				59.3 μM		/	

Table S9. Compounds 271–324 with Immunosuppressive Effects.

Noumber	Compounds	Type	Source	Model 1	Activities IC_{50}	Model 2	Activities IC_{50}	Reference
271	(+)-Adprepyrone B	Fungi	*Talaromyces adpressu*	ConA-induced T-cell proliferation	11.6 μM	/	/	[89]
272	(+)-Adprepyrone D				8.9 μM		/	
273	(–)-Adprepyrone D				9.6 μM		/	
274	(+)-Adprepyrone E				16.8 μM		/	
275	(–)-Adprepyrone E				16.8 μM		/	
276	6-[(*E*)-3-Hydroxyprop-1-enyl]-4-methoxy-5-methyl-2-pyrone				19.8 μM		/	
277	Pinoresinol	Plant	*Epigynum cochinchinensis*	ConA-induced T-cell proliferation	12.5 μM	/	/	[90]
278	Syringaresinol				50 μM		/	
279	Sydoxanthone B	Fungi	*Aspergillus sydowii*	ConA-induced T-cell proliferation	22.53 μM	LPS-induced B-cells proliferation	15.3 μM	[91]
280	13-*O*-acetylsydowinin B				172.7 μM		/	
281	Diaporchromone A	Plant	*Rhizophora mangle*	ConA-induced T-cell proliferation	34 μM	LPS-induced B-cells proliferation	117 μM	[92]
282	Peniphenone	Fungi	*Penicillium sp.* ZJ-SY2	ConA-induced T-cell proliferation	8.1 μM	LPS-induced B-cells proliferation	9.3 μM	[93]

283	Methyl peniphenone	Fungi	*Penicillium sp.* ZJ-SY2	ConA-induced T-cell proliferation	17.5 μM	B-cells proliferation	23.7 μM	[93]
284	Conioxanthone A				8.2 μM		7.5 μM	
285	Methyl-8-hydroxy-6-methyl-9-oxo-9H-xanthene -1-carboxylate				25.7 μM		26.4 μM	
286	Pinselin				5.9 μM		7.5 μM	
287	Sydowinin B				19.2 μM		20.8 μM	
288	Sydowinin A				6.5 μM		7.1 μM	
289	Remisporine B				30.1 μM		32.4 μM	
290	Epiremisporine B				30.8 μM		31.2 μM	
291	Pestaphilone A	Fungi	*Pestalotiopsis oxyanthi*	murine T lymphocyte proliferation stimulated by Con A	9.36 μM	/	/	[94]
292	Pestaphilone B				21.78 μM		/	
293	Pestaphilone C				17.94 μM		/	
294	Pestaphilone D				19.72 μM		/	
295	Pestaphilone E				25.64 μM		/	
296	Pestaphilone F				35.21 μM		/	
297	Podospin A	Fungi	*Podospora* sp	ConA-induced T cell proliferation	10.6 μM	LPS-induced B cell proliferation	10.3 μM	[95]
298	Podospin E				25.1 μM		27.7 μM	
299	Podospin J				18.8 μM		21.9 μM	
300	Podospin L				16.5 μM		14.6 μM	
301	LL-Z1640-1				7.4 μM		6.4 μM	
302	(5*E*)-7-oxozeaenol				6.0 μM		6.2 μM	
303	Cochliomycin D				11.4 μM		6.8 μM	
304	Cochliomycin E				24.1 μM		29.1 μM	
305	Cochliomycin F				15.2 μM		11.8 μM	

306	Dihydrohypothemycin	Fungi	*Podospora* sp	ConA-induced T cell proliferation	8.1 μM		8.5 μM	[95]
307	Aigialomycin D				16.3 μM		18.4 μM	
308	1-naphthaleneheptanoic acid	Fungi	*Tripterygium wilfordii.*	anti-CD3/anti-CD28 stimulated spleen cells	16.51 μM	/	/	[96]
309	Monacolin K				8.75 μM		/	
310	Monacolin L				5.3 μM		/	
311	Monacolin J				6.74 μM		/	
312	Curtachalasin F	Fungi	*Xylaria cf. curta*	ConA-induced T-cell proliferation	70.9 μM	LPS-induced B-cell proliferation	2.4 μM	[75]
313	Curtachalasin H				28.5 μM		72.6 μM	
314	Curtachalasin I				13.3 μM		72.6 μM	
315	Curtachalasin J				21.0 μM		/	
316	Curtachalasin M				31.1 μM		/	
317	Curtachalasin N				62.5 μM		/	
318	Curtachalasin O				12.6 μM		/	
319	Curtachalasin P				16.5 μM		72.3 μM	
320	urtachalasins A				29.0 μM		/	
321	Curtachalasins B				39.7 μM		35.4 μM	
322	Curtachalasins E				22.4 μM		88.0 μM	
323	Ivorenolide B	Plant	*Khaya ivorensis*	LPS-induced B-cell proliferation	7.2 μM	/	/	[97]
324	Efophylin B	Fungi	*Streptomyces malaysiensis*	ConA-induced T-cell proliferation	24.6 μM	/	/	[98]
